# Stereoselective synthesis of tricyclic compounds by intramolecular palladium-catalyzed addition of aryl iodides to carbonyl groups

**DOI:** 10.3762/bjoc.12.118

**Published:** 2016-06-16

**Authors:** Jakub Saadi, Christoph Bentz, Kai Redies, Dieter Lentz, Reinhold Zimmer, Hans-Ulrich Reissig

**Affiliations:** 1 Freie Universität Berlin, Institut für Chemie und Biochemie, Takustrasse 3, D-14195 Berlin, Germany

**Keywords:** 1,2-addition, aryl iodides, ketones, nucleophilic addition, palladium catalysis

## Abstract

Starting from γ-ketoesters with an *o*-iodobenzyl group we studied a palladium-catalyzed cyclization process that stereoselectively led to bi- and tricyclic compounds in moderate to excellent yields. Four X-ray crystal structure analyses unequivocally defined the structure of crucial cyclization products. The relative configuration of the precursor compounds is essentially transferred to that of the products and the formed hydroxy group in the newly generated cyclohexane ring is consistently in *trans*-arrangement with respect to the methoxycarbonyl group. A transition-state model is proposed to explain the observed stereochemical outcome. This palladium-catalyzed Barbier-type reaction requires a reduction of palladium(II) back to palladium(0) which is apparently achieved by the present triethylamine.

## Introduction

**Scheme 1:**
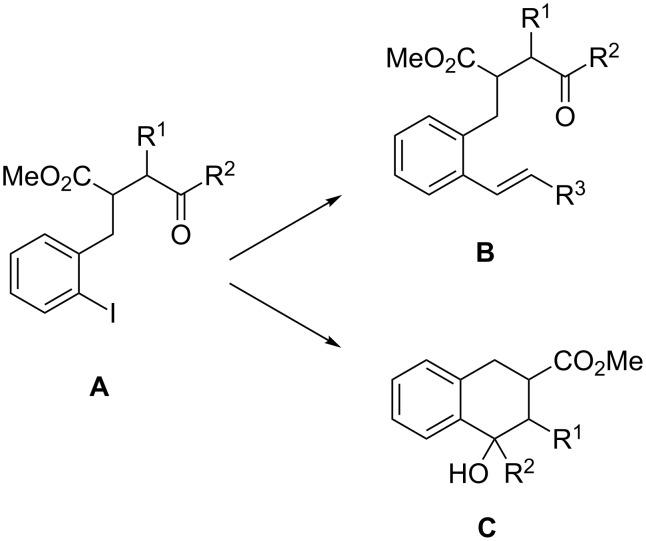
Planned Heck reaction of **A** to compound **B** and serendipitous discovery of the palladium-catalyzed cyclization to products **C**.

For our systematic studies on samarium diiodide promoted cyclizations leading to benzannulated medium-sized rings [1–4] we required starting materials such as alkenyl-substituted compounds **B** (Scheme 1). Obvious precursors for **B** are aryl iodides **A** that smoothly undergo palladium-catalyzed coupling reactions to provide the desired products. However, in one case [**A**: R^1^–R^2^ = (CH_2_)_4_] typical Heck reaction conditions employing styrene as olefin component not only led to the desired styrene derivative **B** but mainly to the cyclized product **C**. If the reaction was performed without the olefin it provided only the tertiary alcohol **C** in reasonable yield [5]. Similar C–C bond forming reactions of aryl halides that involve an insertion of the intermediate aryl palladium species into a carbonyl group are relatively rare (see discussion below). Therefore this serendipitous discovery led us to investigate the reaction in more detail.

## Results

**Scheme 2:**
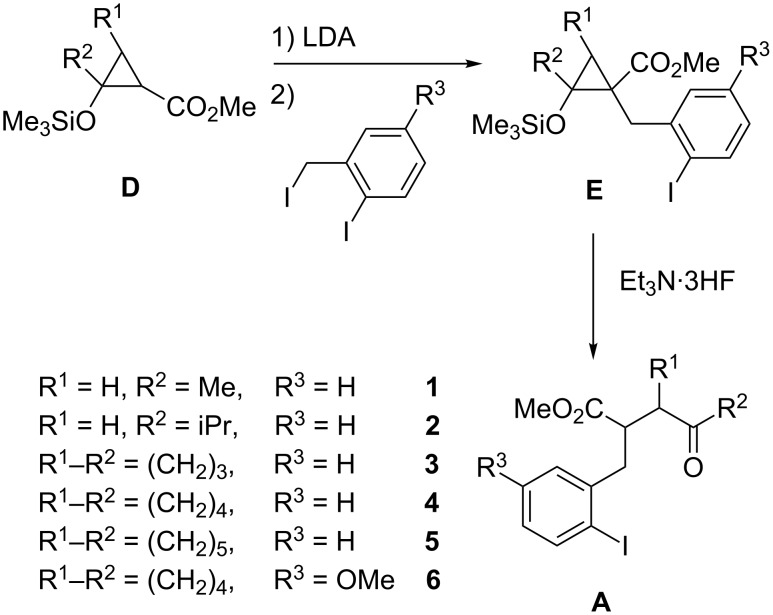
Synthesis of compounds **A** (**1**–**6**) via methyl 2-siloxycyclopropanecarboxylates **D**, their alkylation to **E** and fluoride-induced ring opening.

The required γ-ketoesters **A** bearing the aryl iodide substituent were prepared following our well-established route via 2-siloxycyclopropane carboxylates **D** [6–7] that allows a regioselective introduction of the benzylic substituents at the α-carbon [8–9] to give intermediates **E** (Scheme 2). After fluoride-promoted ring opening [10] the desired precursor compounds **A** (**1**–**6**) were obtained in reasonable overall efficacies (for details see Supporting Information File 1).

**Scheme 3:**
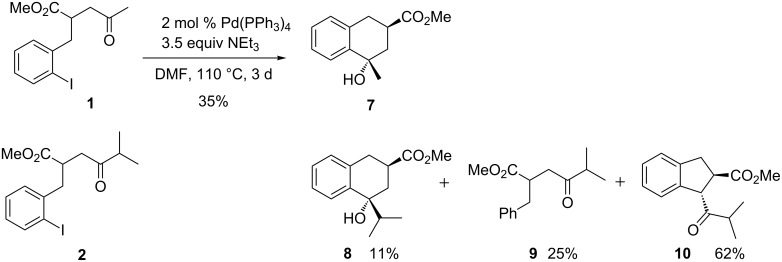
Palladium-catalyzed reactions of methyl ketone **1** to tetralin derivative **7** and of isopropyl-substituted ketone **2** to compounds **8**, **9** and **10**.

We start our report with the palladium-catalyzed reactions of simple alkyl ketones **1** and **2** leading to bicyclic products and then continue with the transformations of cyclic ketones **3**–**6** that led to tricyclic compounds. Methyl ketone **1** provided under the reaction conditions (2 mol % Pd(PPh_3_)_4_, 3.5 equivalents NEt_3_, DMF, 110 °C, 3 d) that had been optimized with compound **4** a moderate yield of the tetralin derivative **7** formed as a single diastereomer (Scheme 3). Although the configurational assignment is ambiguous in this case, the NMR data and the fact that no γ-lactone is formed strongly support the *trans*-arrangement of the two functional groups as depicted. Under similar conditions (5 mol % Pd(PPh_3_)_4_, 90 °C, 3 d) the isopropyl-substituted ketone **2** furnished a mixture of the related *trans*-compound **8** (11%) together with the de-iodinated product **9** (25%) and the indane derivative **10** as major component (62%). The C–C coupling reaction to **8** seems to be hindered in this case, probably due to the steric bulk of the isopropyl group. The formation of indane derivative **10** occurs by an intramolecular enolate arylation, a reaction that has been discovered by our group some years ago [11–12]. Apparently, under the reaction conditions a ketone enolate of **2** reacts with the iodoarene moiety to form the five-membered ring of **10**. The configurational assignments of compounds **7** and **8** are in agreement with those discussed below, where X-ray crystal structure analyses unequivocally confirmed the relative configurations of cyclization products.

**Scheme 4:**
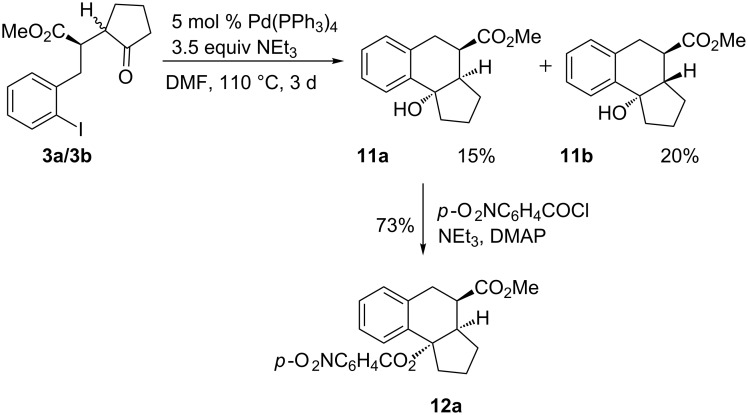
Palladium-catalyzed cyclization of diastereomeric cyclopentanone derivatives **3a/3b** to products **11a** and **11b** and synthesis of *p*-nitrobenzoate **12a**.

**Figure 1:**
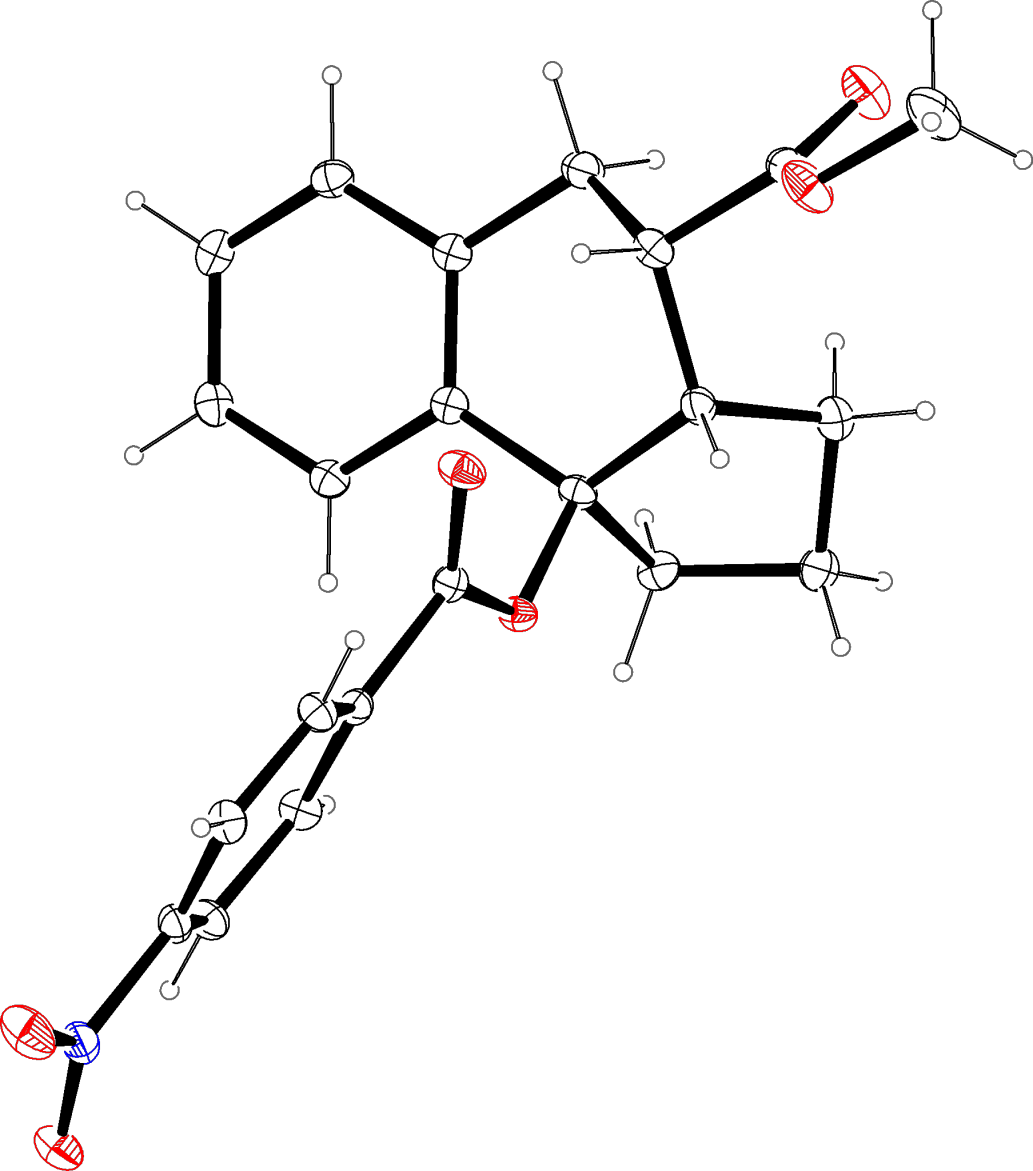
Molecular structure (ORTEP, [14]) of compound **12a** (thermal ellipsoids at 50% probability).

**Scheme 5:**
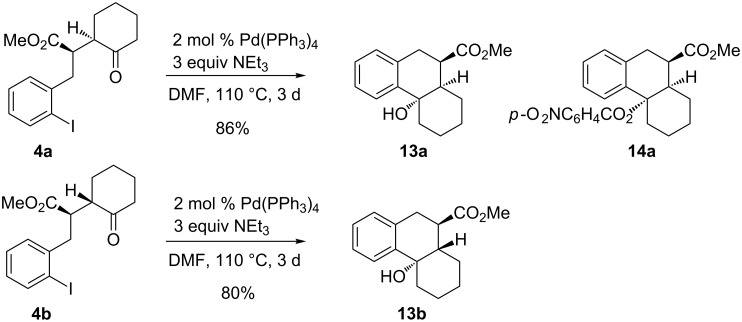
Palladium-catalyzed cyclizations of diastereomeric cyclohexanone derivatives **4a** and **4b** leading stereoselectively to products **13a** and **13b** and structure of *p*-nitrobenzoate **14a**.

**Scheme 6:**
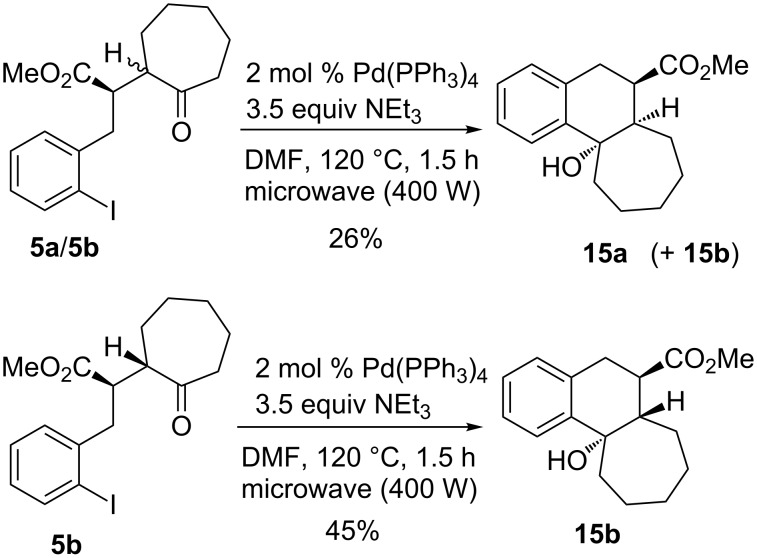
Palladium-catalyzed cyclizations of cycloheptanone derivatives **5a** and **5b** leading to products **15a** and **15b**.

With the cyclic ketones in part considerably higher yields of tricyclic products could be obtained (Schemes 4–6). The cyclopentanone derivative **3** (Scheme 4) had to be used as a mixture of two diastereomers (ca. 2:1) since these were not separable in our hands. Under the standard reaction conditions this ketone furnished a mixture of two diastereomeric tricyclic products **11a** and **11b** in 35% combined yield. The configuration of **11a** was unequivocally determined by an X-ray crystal structure analysis of the corresponding *p*-nitrobenzoate **12a** obtained by esterification of the tertiary alcohol under standard conditions (Scheme 4, Figure 1) [13]. The configuration of the second product **11b** is only tentatively assigned as depicted since the available data do not allow an unambiguous determination. Considering the result obtained with the cycloheptanone derivative where two diastereomers could be assigned by X-ray crystal structure analyses make the proposed *trans*-annulation of the five- and six-membered rings fairly likely.

**Figure 2:**
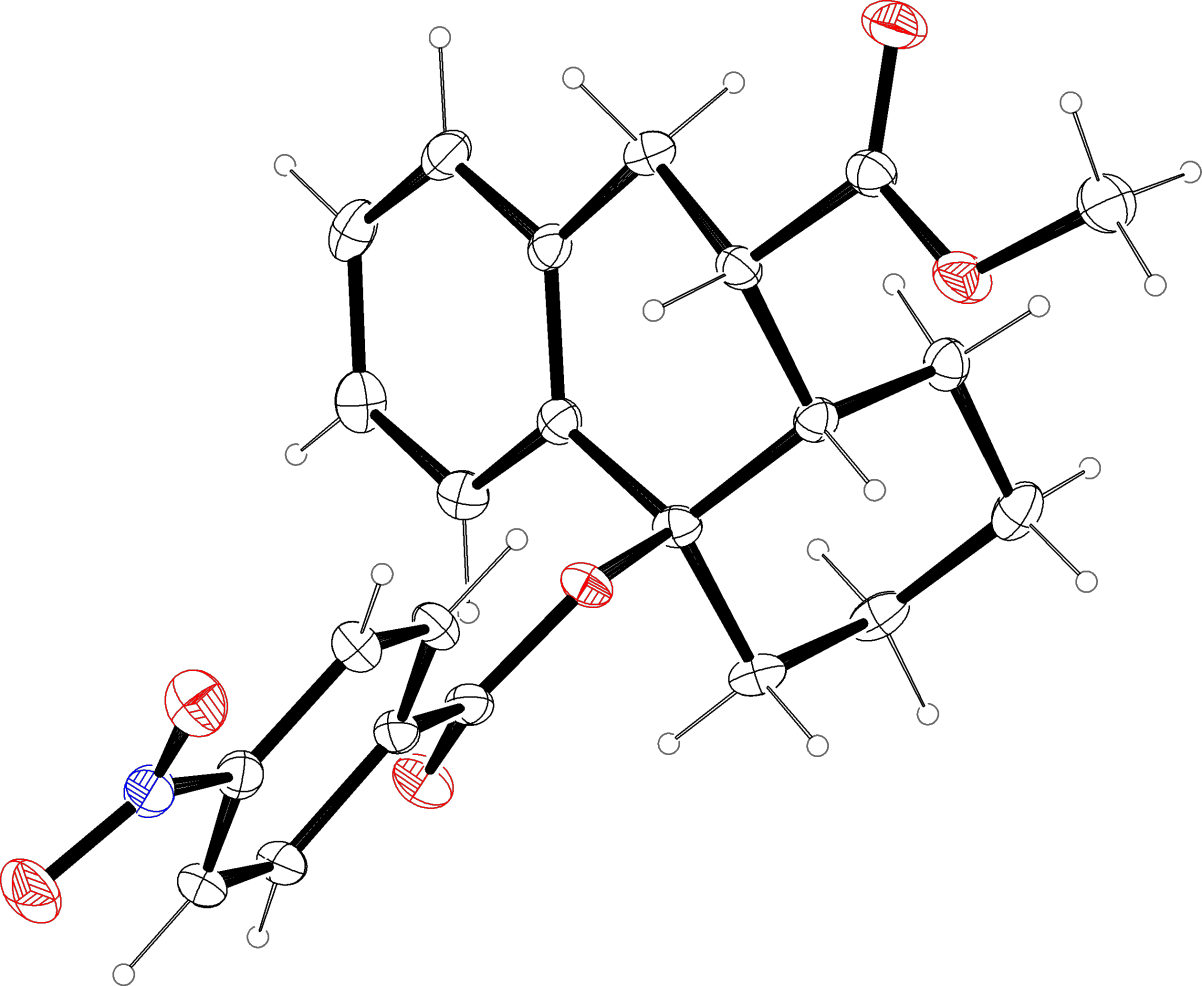
Molecular structure (ORTEP, [14]) of compound **14a** (thermal ellipsoids at 50% probability).

The palladium-catalyzed cyclization discussed in this report was discovered with cyclohexanone derivative **4** and we therefore tried to optimize the reaction conditions with this substrate. After extensive studies investigating different palladium catalysts, bases, additives, temperatures (with and without microwave) and reaction times we found the recorded conditions to be most reliable. A mixture of the two diastereomers **4a**/**4b** (ca. 1:1) furnished the two isomeric cyclization products **13a** and **13b** in varying yields and in several experiments only compound **13a** was isolated. Fortunately, diastereomers **4a** and **4b** could be separated by conventional column chromatography and hence a detailed analysis of the stereochemical features of this transformation was possible. Configurationally homogeneous compound **4a** furnished diastereomerically pure cyclization product **13a** in excellent yield, whereas epimer **4b** was converted into product **13b** in 80% yield, again formed as a single isomer (Scheme 5). An unequivocal configurational assignment of **13a** was possible by the X-ray crystal structure analysis of its corresponding *p*-nitrobenzoate **14a** (Figure 2) [15]. The configuration of compound **13b** was tentatively assigned in analogy to its lower and higher homologs.

**Figure 3:**
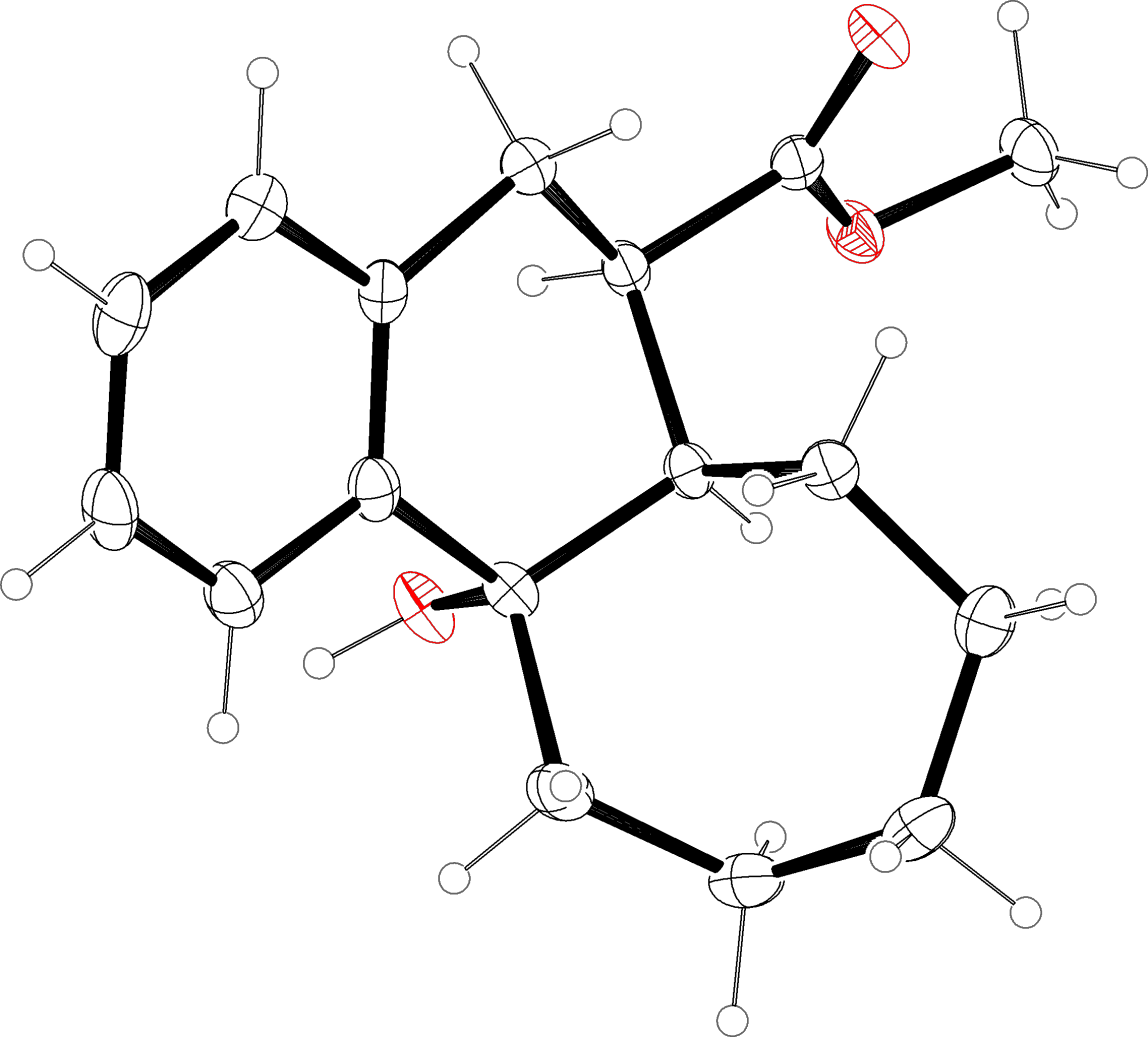
Molecular structure (ORTEP, [14]) of compound **15a** (thermal ellipsoids at 50% probability).

**Figure 4:**
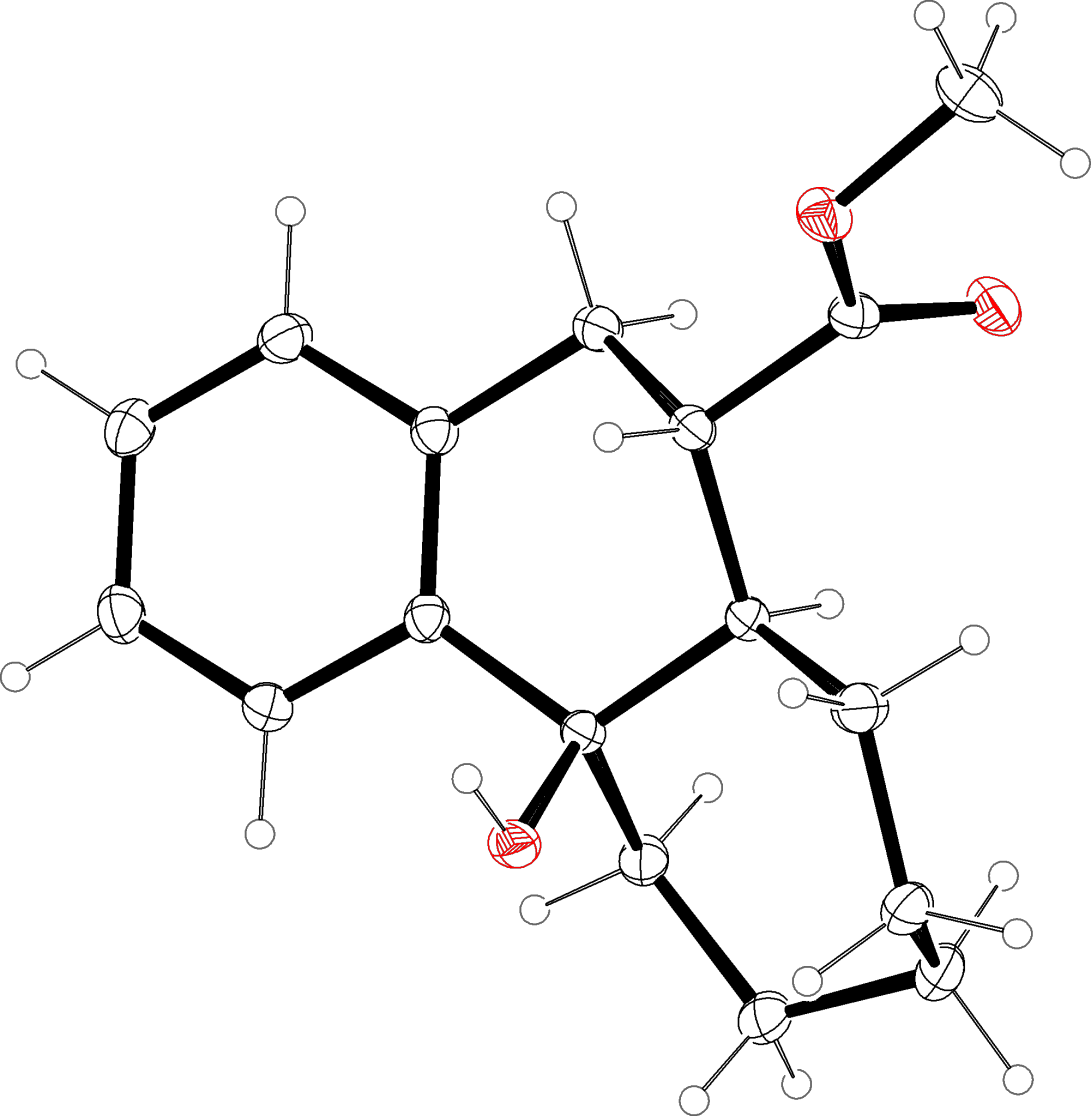
Molecular structure (ORTEP [14]) of compound **15b** (thermal ellipsoids at 50% probability).

With cycloheptanone derivatives **5a**/**b** less efficient cyclizations were observed. The mixture of diastereomers **5a**/**5b** (ca. 1:1) provided a mixture of compounds containing the two isomers **15a** and **15b**, from which pure **15a** could be isolated (Scheme 6). A diastereomerically enriched sample of **5b** afforded compound **15b** in 45% yield as a single isomer. These results were obtained employing microwave irradiation (400 W) that allowed considerably shorter reaction times, however, the yields were not strongly influenced by this modification. From both product diastereomers crystals suitable for X-ray crystal structure analyses could be obtained (Figure 3 and Figure 4) [16–17]. Again, the configurations of the precursors are reflected in the product structure. Compound **5b** provided product **15b** with *trans*-annulation of the two rings and we assume that compound **15a** with *cis*-annulated rings was formed selectively from precursor **5a**.

**Scheme 7:**
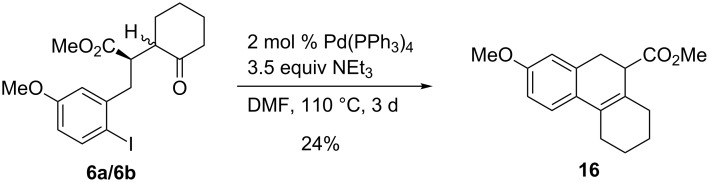
Palladium-catalyzed cyclization of *p*-methoxy-substituted aryl iodide **6a**/**6b** to compound **16**.

We also briefly studied the palladium-catalyzed cyclization of *p*-methoxy-substituted aryl iodide **6a**/**b** that led under the standard conditions to a mixture containing compound **16** (Scheme 7). We cannot exclude that other regioisomers or even primarily formed tetralin derivatives are in the crude product mixture, but we isolated only compound **16** in pure form in 24% yield. Not surprisingly, the *p*-methoxy substituent favored the elimination of water from the primary addition product to generate the central double bond of **16**.

## Discussion

**Scheme 8:**
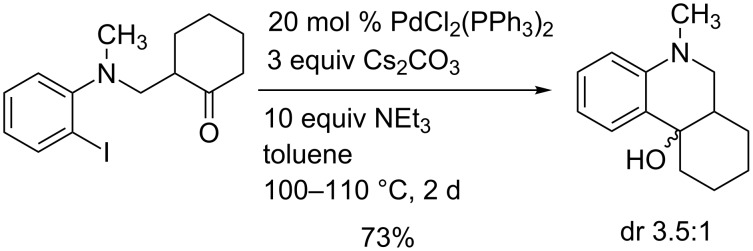
Typical palladium-catalyzed cyclization of an *o*-iodoaniline derivative to a tricyclic tertiary alcohol as reported by Solé et al. [23].

The nucleophilic addition of organometallics such as lithium, magnesium or zinc organic compounds to carbonyl groups leading to alcohols is an important standard operation in organic synthesis providing high stereoselectivities in many cases. The in situ generation of the nucleophilic species from the corresponding organic halides in the presence of the carbonyl compound is also known, e.g., the Barbier reaction employing magnesium. The related transformation described in this report very likely involves an arylpalladium species as nucleophile that is in situ generated from the aryl iodide moiety. Similar palladium-catalyzed processes (palladium Barbier reactions) are relatively rare (for a review, see [18]). Early studies were reported by Y. Yamamoto et al. [19] and this group also published examples involving an alkyne palladation step to vinyl palladium intermediates that are able to undergo additions to carbonyl groups [20–21]. Very extensive investigations with a variety of *o*-haloaniline derivatives as precursors have been reported by the group of Solé, Bonjoch and Fernández [22–23]. They also analyzed this reaction and the competing enolate arylation by computational studies [24–25] (for a review, see [26]). Singular contributions employing different systems were contributed by other groups [27–29]. Scheme 8 shows a typical example of Solé et al. [23], related to our systems, furnishing a tricyclic compound in good yield with moderate diastereoselectivity.

**Scheme 9:**
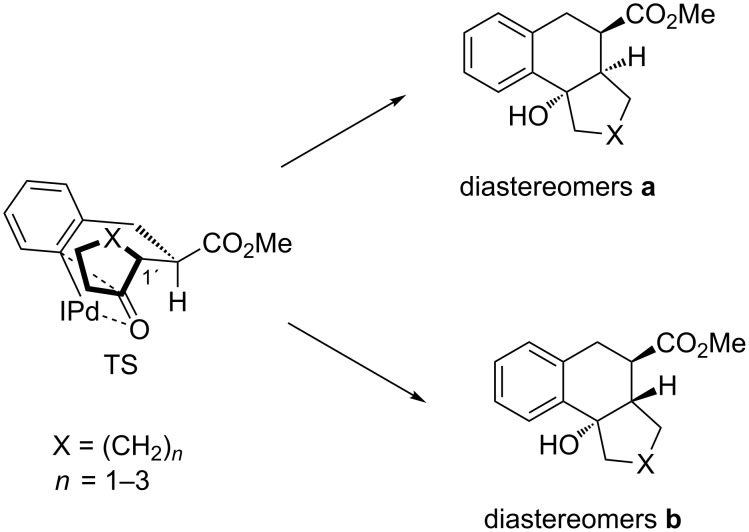
Proposed transition state (TS) explaining the stereoselective formation of cyclization products.

The transformations described in the present report are highly stereoselective (we hesitate to use the term stereospecific here since the mass balances are often too low to rigorously exclude the formation of other diastereomers) since the configuration of the cyclic precursor ketones **3**–**5** is transferred to that of the cyclization products as shown in Schemes 4–6. Surprisingly, the presence of excess of the base triethylamine and the fairly long reaction times do not lead to noticeable epimerization of the precursor γ-ketoesters that would lead to an erosion of the observed stereoselectivity. In all examples the methoxycarbonyl group and the hydroxy group are arranged *trans* to each other irrespective of the configuration of the third stereogenic center at the bridgehead. A transition-state model that rationalizes this observation is depicted in Scheme 9. We propose a four-center interaction of the carbonyl moiety with the carbon–palladium bond in the transition state (TS) and due to this highly ordered arrangement only a boat-like transition state with a pseudo-equatorial position of the methoxycarbonyl group seems to be possible. The rigid benzene backbone further restricts the flexibility of the system. This model explains the observed *trans*-arrangement of the methoxycarbonyl group and the hydroxy group leading to diastereomers **a**, if the hydrogen at C-1’ is in a pseudo-equatorial position and diastereomers **b** if this atom occupies a pseudo-axial position. This transition state is in accordance with the model that has been proposed on the basis of DFT calculations by Solé and Fernández [24–26].

**Scheme 10:**
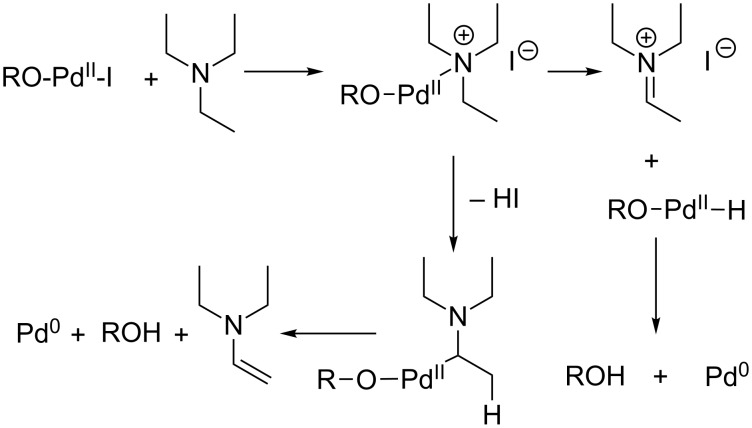
Possible mechanism of the reduction of palladium(II) to palladium(0) by triethylamine (additional ligands at palladium are not depicted for clarity of the presentation).

The intermediate arylpalladium species is certainly generated by the standard oxidative insertion of palladium(0) into the C–I bond of the aryl iodide moiety. After the C–C bond formation leading to the bi- or tricyclic products, palladium(II) has to be reduced back to a palladium(0) species in order to allow a catalytic use of the metal. It is well known that several reagents (alkenes or alcohols [19]) are able to achieve this reduction. We therefore assume that triethylamine is the reducing reagent under the conditions employed in this study [30]. Possible mechanisms for this process leading to the formation of an iminium intermediate or a dehydrogenation of the amine are depicted in Scheme 10.

## Conclusion

We have found new examples of intramolecular palladium-catalyzed nucleophilic additions of aryl iodides to alkyl ketones. These additions proceed in the presence of only 2–5 mol % Pd(PPh_3_)_4_ and afford bi- and tricyclic compounds with excellent stereoselectivity and in moderate to very good efficacy. The low mass balance observed in several cases may be due to subsequent reactions such as simple de-iodination of the precursor compounds or elimination of water in the products. However, in general none of these byproducts has been isolated. For compound **2** the bulky isopropyl group slows down the addition to the carbonyl group and an enolate arylation was observed instead as major reaction pathway. Although the scope of the discovered aryl iodide addition to carbonyl groups may be limited it is attractive since only low catalyst loadings are required and interesting products are formed with high stereoselectivity.

## Supporting Information

File 1 Characterization data and copies of ^1^H and ^13^C NMR spectra.
