# Supplementary material for: Stereoselective synthesis of tricyclic compounds by intramolecular palladium-catalyzed addition of aryl iodides to carbonyl groups
Source: Beilstein J Org Chem. 2016 Jun 16;12:1236–42. doi: 10.3762/bjoc.12.118 (PMC4979759; doi:10.3762/bjoc.12.118)
Supplement: File 1 — Characterization data and copies of 1H and 13C NMR spectra. [file Beilstein_J_Org_Chem-12-1236-s001.pdf]

Supporting Information

for

Stereoselective synthesis of tricyclic compounds by intramolecular palladium-catalyzed addition of aryl iodides to carbonyl groups

Jakub Saadi[1], Christoph Bentz[1], Kai Redies[1], Dieter Lentz[1], Reinhold Zimmer[1], Hans-Ulrich Reissig*[1]

Address: [1]Freie Universität Berlin, Institut für Chemie und Biochemie, Takustrasse 3, D-14195 Berlin, Germany

Email: Hans-Ulrich Reissig - hreissig@chemie.fu-berlin.de

* Corresponding author

Characterization data and copies of ^{1}H and ^{13}C NMR spectra

Contents

1) General information S2

2) Experimental procedures S2

3) References. S12

4) Copies of ^{1}H and ^{13}C NMR spectra S13

1) General information

Reactions were performed under argon in a pressure tube. Microwave-assisted reactions were carried out in amicrowave oven ("microChemist", MLS GmbH). Triethylamine was distilled from calcium hydride and stored under argon over KOH. *N,N*-Dimethylformamide was purchased in p.A. purity grade and stored under argon over activated 4 Å molecular sieves. Dichloromethane was purified with the MB SPS-800-dry solvent system. Hexanes were distilled from CaH_2, ethyl acetate was distilled from K_2CO_3 and $CaCl_2$. Products were purified by flash chromatography on silica gel (230–400 mesh, Merck) or on alumina (activity grade III, Macherey & Nagel).

^{1}H NMR [$CHCl_3$ (δ = 7.26 ppm) or TMS (δ = 0.00 ppm) as internal standards] and ^{13}C NMR spectra [$CDCl_3$ (δ = 77.16 ppm) as internal standard] were recorded on Bruker (AC 500, AVIII 700) and JEOL (ECX 400, Eclipse 500) instruments in $CDCl_3$ solution. Integrals are in accordance with assignments; coupling constants are given in Hz. For detailed peak assignments 2D spectra were measured (COSY, HMQC, HMBC). IR spectra were measured with an FTIR spectrometer Nicolet 5 SXC or with a Nexus FT-IR equipped with a Nicolet Smart DuraSamplIR ATR. MS and HRMS analyses were performed with Varian Ionspec QFT-7 (ESI-FT ICRMS), Agilent 6210 (ESI–TOF) or Finnigan MAT 711 (EI, 80 eV, 8 kV) instruments. Elemental analyses were carried out with CHN-Analyzer Vario EL or Vario EL III instruments. Melting points were measured with a Reichert apparatus (Thermovar) and are uncorrected.

2) Syntheses of starting materials

The preparation of compounds **1** [1], **2** [2], **4** [3] and **5** [3] has been reported earlier.

Methyl 6-(2-iodobenzyl)-1-(trimethylsiloxy)bicyclo[3.1.0]hexane-6-carboxylate:

This compound was prepared analogously to the published procedure [1-3]. After flash chromatography (alumina, hexanes to hexanes/ethyl acetate 9:1) it was obtained as colorless oil in 85% yield.

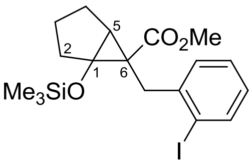

^{1}H NMR ($CDCl_3$, 500 MHz): δ = 0.25 (s, 9 H, $SiMe_3$), 0.99-1.10 (m, 1 H, 3-H), 1.62-1.64 (m, 1 H, 5-H), 1.72-1.79, 1.97-2.01, 2.05-2.12 (3 m, 3 × 1 H, 2-H, 3-H), 2.13 (ddd, *J* = 10.2, 10.3, 12.8 Hz, 1 H, 4-H), 2.24 (ddd, *J* = 1.8, 8.9, 12.8 Hz, 1 H, 4-H), 3.03 (d, *J* = 16.4 Hz, 1 H, ArCH_2), 3.27 (d, *J* = 16.4 Hz, 1 H, ArCH_2), 3.55 (s, 3 H, CO_2Me), 6.87-6.90, 7.28-7.31, 7.53-7.55, 7.80-7.82 (4 m, 4 × 1 H, Ar) ppm.

^{13}C NMR ($CDCl_3$, 126 MHz): δ = 1.2 (q, $SiMe_3$), 22.4, 22.8 (2 t, C-2, C-3), 33.8 (t, C-4), 36.4 (d, C-5), 37.3 (s, C-6), 42.4 (t, ArCH_2), 73.2 (s, C-1), 101.5 (s, C-I), 128.0, 128.2, 129.6, 139.4, 141.9 (4 d, s, Ar), 51.7, 171.7 (q, s, CO_2Me) ppm.

IR (neat): $\tilde{\nu}$ = 3060-2840 (=C-H, C-H), 1725 (C=O) cm^{-1}.

EA: $C_{18}H_{25}IO_3Si$ (444.4) calcd. (%): C 48.65, H 5.67; found (%): C 48.13, H 5.49.

Methyl 3-(2-iodophenyl)-2-(2-oxocyclopentyl)propanoate (3a,b):

This compound was prepared analogously to the published procedure [1-3]. After column chromatography (silica gel, hexanes/ethyl acetate 4:1) **3** was obtained in 78% yield as a mixture of two diastereomers (dr = 69:31) as colorless oil.

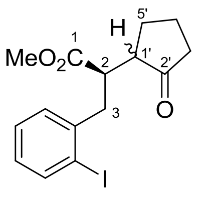

3a/b

^{1}H NMR ($CDCl_3$, 500 MHz): major isomer, δ = 1.69-1.90, 2.03-2.37 (2 m, 2 H, 5 H, 1'-H, 3'-H, 4'-H, 5'-H), 2.98 (dd, *J* = 8.7, 14.1 Hz, 1 H, 3-H), 3.29 (dd, *J* = 6.9, 14.1 Hz, 1 H, 3-H), 3.47 (ddd, *J* = 4.4, 6.9, 8.7 Hz, 1 H, 2-H), 3.62 (s, 3 H, CO_2Me), 6.89-6.93, 7.18-7.28, 7.80-7.84 (3 m, 1 H, 2 H, 1 H, Ar) ppm; minor isomer, δ = 1.69-1.90, 2.03-2.37 (2 m, 2 H, 5 H, 1'-H, 3'-H, 4'-H, 5'-H), 2.97 (ddd, *J* = 6.6, 7.5, 9.0 Hz, 1 H, 2-H), 3.05 (dd, *J* = 9.0, 13.3 Hz, 1 H, 3-H), 3.09 (dd, *J* = 6.6, 13.3 Hz, 1 H, 3-H), 3.57 (s, 3 H, CO_2Me), 6.89-6.93, 7.18-7.28, 7.80-7.84 (3 m, 1 H, 2 H, 1 H, Ar) ppm.

^{13}C NMR ($CDCl_3$, 126 MHz): major isomer, δ = 20.7, 26.2 (2 t, C-4', C-5'), 38.1 (t, C-3'), 40.3 (t, C-3), 44.9 (d, C-2), 49.3 (d, C-1'), 100.9 (s, C-I), 128.4, 128.6, 130.1, 140.0, 141.6 (4 d, s, Ar), 51.9, 173.3 (q, s, CO_2Me), 218.9 (s, C-2') ppm; minor isomer, δ = 20.8, 28.0 (2 t, C-4', C-5'), 38.2 (t, C-3'), 41.4 (t, C-3), 46.2 (d, C-2), 50.2 (d, C-1'), 100.6 (s, C-I), 128.3, 128.6, 130.5, 139.8, 141.3 (4 d, 1 s, Ar), 51.7, 174.5 (q, s, CO_2Me), 218.5 (s, C-2') ppm.

IR (neat): $\tilde{\nu}$ = 3050-2875 (=C-H, C-H), 1725 (br, C=O) cm^{-1}.

EA: $C_{15}H_{17}IO_3$ (372.2) calcd. (%): C 48.40, H 4.60; found (%): C 48.21, H 4.47.

Methyl 7-(2-iodo-5-methoxybenzyl)-1-(trimethylsiloxy)bicyclo[4.1.0]heptane-7-carboxylate:

This compound was prepared analogously to the published procedure [1-3]. After column chromatography (alumina, hexanes, then hexanes/ethyl acetate 9:1 to 4:1) it was obtained as colorless oil in 55% yield.

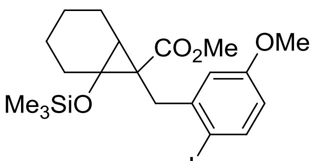

^{1}H NMR ($CDCl_3$, 500 MHz): δ = 0.23 (s, 9 H, $SiMe_3$), 1.12-1.20, 1.24-1.32, 1.35-1.47, 1.96-2.04, 2.06-2.14 (5 m, 2 x 1 H, 3 x 2 H, 2-H, 3-H, 4-H, 5-H), 1.28 (dd, *J* = 8.7, 1.9 Hz, 1 H, 6-H), 2.94 (d, *J* = 16.8 Hz, 1 H, ArC*H$_2$*), 3.28 (d, *J* = 16.8 Hz, 1 H, ArC*H$_2$*), 3.60 (s, 3 H, CO_2Me), 3.78 (s, 3 H, OMe), 6.50 (dd, *J* = 8.7, 3.1 Hz, 1 H, Ar), 7.16 (d, *J* = 3.1 Hz, 1 H, Ar), 7.64 (d, *J* = 8.7 Hz, 1 H, Ar) ppm.

^{13}C NMR ($CDCl_3$, 126 MHz): δ = 1.4 (q, $SiMe_3$), 19.3, 20.9, 21.1, 30.5 (4 t, C-2, C-3, C-4, C-5), 30.1 (d, C-6), 36.6 (s, C-7), 43.5 (t, $ArCH_2$), 55.5 (q, OMe), 62.0 (s, C-1), 89.6 (s, C-I), 114.5, 114.6, 139.4, 143.4 (3 d, s, Ar), 159.9 (s, C-OMe), 51.5, 171.7 (q, s, CO_2Me) ppm.

IR (neat): ṽ = 3000 (=C-H), 2950, 2840 (C-H), 1720 (C=O), 1590, 1560 (C=C), 1080 (C-I) cm^{-1}.
HRMS (ESI-TOF): $C_{20}H_{29}IO_4Si$ Na^+ calcd.: 511.0772; found: 511.0764.

Methyl 3-(2-iodo-5-methoxyphenyl)-2-(2-oxocyclohexyl)propanoate (6a,b):
Compound **6** was prepared analogously to the published procedure [1-3]. After column chromatography (silica gel, hexanes/ethyl acetate 9:1 to 4:1) afforded 26% of isomer **6a** as colorless oil, 2% of isomer **6b** as colorless solid, and 63% of a 20:80 mixture of **6a/6b** as colorless solid.

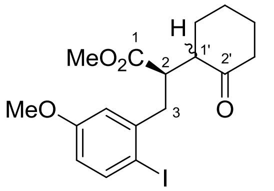

6a/b

Isomer 6a
^{1}H NMR ($CDCl_3$, 500 MHz): δ = 1.64-1.79, 1.88-1.94, 2.01-2.07, 2.08-2.14 (4 m, 3 H, 3 x 1 H, 4'-H, 5'-H, 6'-H), 2.31-2.38 (m, 1 H, 3'-H), 2.50 (dtd, *J* = 13.8, 4.3, 1.5 Hz, 1 H, 3'-H), 2.60-2.66 (m, 1 H, 1'-H), 2.96 (m, 2 H, 3-H), 3.34 (dt, *J* = 8.1, 7.3 Hz, 1 H, 2-H), 3.57 (s, 3 H, CO_2Me), 3.76 (s, 3 H, OMe), 6.51 (dd, *J* = 8.7, 3.0 Hz, 1 H, Ar), 6.81 (d, *J* = 3.0 Hz, 1 H, Ar), 7.65 (d, *J* = 8.7 Hz, 1 H, Ar) ppm.
^{13}C NMR ($CDCl_3$, 126 MHz): δ = 24.6, 27.6, 30.8 (3 t, C-4', C-5', C-6'), 40.7 (t, C-3), 42.1 (t, C-3'), 45.1 (d, C-2), 52.3 (d, C-1'), 55.3 (q, OMe), 89.0 (s, C-I), 114.6, 115.8, 140.0, 142.9 (3 d, s, Ar), 159.8 (s, C-OMe), 51.5, 174.1 (q, s, CO_2Me), 210.7 (s, C-2') ppm.
IR (neat): ṽ = 3000, 2940, 2860 (C-H), 1730, 1710 (C=O), 1590, 1570 (C=C), 1045 (C-I) cm^{-1}.
HRMS (ESI-TOF): $C_{17}H_{21}IO_4$ Na^+ calcd.: 439.0382; found: 439.0390.
EA: $C_{17}H_{21}IO_4$ (416.3) calcd. (%): C 49.05, H 5.09; found (%): C 49.11, H 5.09.

Isomer 6b
M.p.: 85-87 °C
^{1}H NMR ($CDCl_3$, 500 MHz): δ = 1.59-1.77, 1.95-2.01, 2.08-2.13 (3 m, 3 H, 2 x 1 H, 4'-H, 5'-H, 6'-H), 2.32-2.45 (m, 3 H, 3'-H, 6'-H), 2.77 (dd, *J* = 13.2, 10.8 Hz, 1 H, 3-H), 2.80-2.85 (m, 1 H, 1'-H), 2.94 (ddd, *J* = 10.8, 8.4, 4.7 Hz, 1 H, 2-H), 3.03 (dd, *J* = 13.2, 4.7 Hz, 1 H, 3-H), 3.47 (s, 3 H, CO_2Me), 3.75 (s, 3 H, OMe), 6.52 (dd, *J* = 8.7, 3.0 Hz, 1 H, Ar), 6.70 (d, *J* = 3.0 Hz, 1 H, Ar), 7.65 (d, *J* = 8.7 Hz, 1 H, Ar) ppm.
^{13}C NMR ($CDCl_3$, 126 MHz): δ = 25.2, 27.5, 31.3 (3 t, C-4', C-5', C-6'), 40.8 (t, C-3), 42.0 (t, C-3'), 45.3 (d, C-2), 52.9 (d, C-1'), 55.3 (q, OMe), 88.9 (s, C-I), 114.6, 115.8, 139.9, 142.5 (3 d, s, Ar), 159.8 (s, C-OMe), 51.3, 175.3 (q, s, CO_2Me), 211.0 (s, C-2') ppm.
IR (ATR): ṽ = 3020 (=C-H), 2940, 2860 (C-H), 1725, 1690 (C=O), 1590, 1560 (C=C), 1040 (C-I) cm^{-1}.
HRMS (ESI-TOF): calcd. for $C_{17}H_{21}IO_4$ Na^+: 439.0382; found: 439.0384.
EA: $C_{17}H_{21}IO_4$ (416.3) calcd. (%): C 49.05, H 5.09; found (%): C 49.16, H 5.01.

General procedure 1, cyclization experiments:

All reactions were performed in a pressure tube under argon. The corresponding aryl iodide and $Pd(PPh_3)_4$ were dissolved in DMF (4 mL/mmol of aryl iodide) and triethylamine (3.5 equivalents). The mixture was heated to 110 °C (in part to 120 °C applying microwave, 400 Watt) for the indicated time. After cooling to room temperature, ethyl acetate and water were added and the two phases were separated. The aqueous phase was extracted with ethyl acetate (3×) and the combined organic phases were extracted with brine and dried (Na_2SO_4) to give the crude product that was further purified as indicated in the individual experiments.

General procedure 2, syntheses of *p*-nitrobenzoates:

The corresponding cyclization product, *p*-nitrobenzoyl chloride (1.05 equivalents), DMAP (0.75 equivalents) were dissolved in CH_2Cl_2 (10 mL/mmol substrate) and triethylamine (1.0 equivalents) was added. The mixture was stirred at room temperature for 36 h. Water was added to this mixture and the aqueous phase was extracted with CH_2Cl_2 (3×). The combined organic phases were washed with brine and dried (Na_2SO_4). After filtration the solvent was removed to obtain the product in sufficient purity.

Methyl (2*RS*,4*SR*)-4-hydroxy-4-methyl-1,2,3,4-tetrahydronaphthalene-2-carboxylate (7):

According to the **GP1**: compound **1** (108 mg, 0.31 mmol), $Pd(PPh_3)_4$ (7 mg, 6 µmol), NEt_3 (102 mg, 1.01 mmol), DMF (1.5 mL), 110 °C, 3 d. Column chromatography (silica gel, hexanes/ethyl acetate 4:1 to 1:1) provided 24 mg (35%) of **7** as colorless oil.

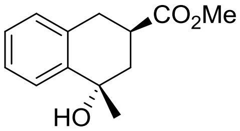

7

^{1}H NMR ($CDCl_3$, 700 MHz): δ = 1.66 (s, 3 H, Me), 1.83 (s_{br}, 1 H, OH), 1.92 (dd, *J* = 13.7, 12.7 Hz, 1 H, 3-H), 2.33 (ddd, *J* = 13.7, 2.8, 2.1 Hz, 1 H, 3-H), 2.93-2.96 (m, 1 H, 1-H), 3.04 (ddd, *J* = 12.7, 4.5, 2.8 Hz, 1 H, 2-H), 3.09 (dddd, *J* = 15.4, 4.5, 2.1, 0.5 Hz, 1 H, 1-H), 3.74 (s, 3 H, CO_2Me), 7.14 (d, *J* = 7.7 Hz, 1 H, Ar), 7.22 (td, *J* = 7.4, 1.4 Hz, 1 H, Ar), 7.25 (t, *J* ≈ 7.4 Hz, 1 H, Ar), 7.55 (dd, *J* = 7.7, 1.4 Hz, 1 H, Ar) ppm.

^{13}C NMR ($CDCl_3$, 176 MHz): δ = 30.0 (q, Me), 32.7 (t, C-1), 36.3 (d, C-2), 41.6 (t, C-3), 69.7 (s, C-4), 126.0, 127.0, 127.8, 129.2, 134.6, 140.4 (4 d, 2 s, Ar), 51.8, 175.7 (q, s, CO_2Me) ppm.

IR (neat): $\tilde{\nu}$ = 3470 (O-H), 3060, 3020 (=C-H), 2950, 2850 (C-H), 1730 (C=O) cm^{-1}.

HRMS (ESI-TOF): $C_{13}H_{16}O_3$ Na^+ calcd.: 243.0992; found: 243.0984.

EA: $C_{13}H_{16}O_3$ (220.3) calcd. (%): C 70.89, H 7.32; found (%): C 70.71, H 7.15.

Methyl (2*RS*,4*RS*)-4-hydroxy-4-isopropyl-1,2,3,4-tetrahydronaphthalene-2-carboxylate (8): According to the **GP1**: compound **2** (200 mg, 0.535 mmol), $Pd(PPh_3)_4$ (30 mg, 27 µmol), NEt_3 (18 mg, 1.78 mmol), DMF (4 mL), 90 °C, 3 d. Column chromatography (silica gel, hexanes/ethyl acetate 4:1 to 1:1) provided 14 mg (11%) of **8**, 33 mg (25%) of **9** and 82 mg (62%) of **10** as colorless oils.

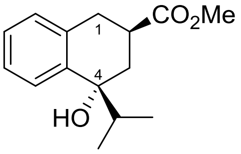

8

^{1}H NMR ($CDCl_3$, 500 MHz): δ = 0.60, 1.13 (2 d, J = 6.9 Hz, 2 × 3 H, CH*Me*$_2$), 1.84 (dd, J = 13.9, 12.3 Hz, 1 H, 3-H), 1.95 (s, 1 H, OH), 2.17 (ddd, J = 13.9, 2.7, 2.4 Hz, 1 H, 3-H), 2.47 (sept, J = 6.9 Hz, 1 H, C*H*Me$_2$), 2.81 (dd, J = 14.5, 12.7 Hz, 1 H, 1-H), 2.88 (dddd, J = 12.7, 12.3, 2.7, 2.6 Hz, 1 H, 2-H), 2.98 (ddd, J = 14.5, 2.6, 2.4 Hz, 1 H, 1-H), 3.73 (s, 3 H, CO_2Me), 7.10-7.25, 7.48-7.49 (2 m, 3 H, 1 H, Ar) ppm.
^{13}C NMR ($CDCl_3$, 126 MHz): δ = 16.3, 18.6 (2 q, CH*Me*$_2$), 33.2, 33.3 (2 t, C-1, C-3), 36.6 (d, C-2), 37.9 (d, C*H*Me$_2$), 75.0 (s, C-4), 125.8, 127.2, 127.5, 129.2, 135.9, 140.4 (4 d, 2 s, Ar), 51.9, 176.1 (q, s, CO_2Me) ppm.
IR (neat): $\tilde{\nu}$ = 3490 (br, O-H), 3060-2845 (=C-H, C-H), 1735 (C=O) cm^{-1}.
MS (EI = 70 eV): *m/z* (%) = 248 (1) $[M]^+$, 217 (4), 242 (6), 205 (100), 173 (32), 145 (62).
EA: $C_{15}H_{20}O_3$ (248.3) calcd. (%): C 72.55, H 8.12; found (%): C 72.47, H 7.75.

Methyl 2-benzyl-5-methyl-4-oxohexanoate (9):

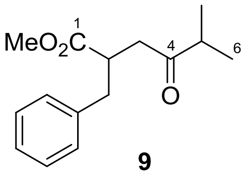

9

^{1}H NMR ($CDCl_3$, 500 MHz): δ = 1.03, 1.07 (2 d, J = 6.9 Hz, 2 × 3 H, 5-Me, 6-H), 2.47 (dd, J = 18.0, 4.6 Hz, 1 H, 3-H), 2.53 (sept, J = 6.9 Hz, 1 H, 5-H), 2.73 (dd, J = 13.6, 8.7 Hz, 1 H, PhC*H*$_2$), 2.87 (dd, J = 18.0, 8.9 Hz, 1 H, 3-H), 3.02 (dd, J = 13.6, 6.3 Hz, 1 H, PhC*H*$_2$), 3.18 (dddd, J = 8.9, 8.7, 6.3, 4.6 Hz, 1 H, 2-H), 3.64 (s, 3 H, CO_2Me), 7.14-7.30 (m, 5 H, Ar) ppm.
^{13}C NMR ($CDCl_3$, 126 MHz): δ = 18.1, 18.3 (2 q, 5-Me, C-6), 37.8 (t, Ph*C*H$_2$), 40.9 (d, C-5), 41.0 (t, C-3), 41.9 (d, C-2), 126.7, 128.6, 129.0, 138.6 (3 d, s, Ar), 51.9, 175.3 (q, s, CO_2Me), 212.7 (s, C-4) ppm.
IR (neat): $\tilde{\nu}$ = 3085-2875 (=C-H, C-H), 1735, 1715 (C=O) cm^{-1}.
EA: $C_{15}H_{20}O_3$ (248.3) calcd. (%): C 72.55, H 8.12; found (%): C 72.27, H 8.03.

Methyl *trans*-1-isobutyrylindane-2-carboxylate (10):

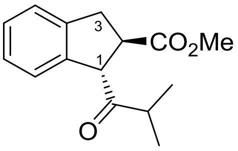

10

^{1}H NMR ($CDCl_3$, 500 MHz): δ = 1.16, 1.22 (2 d, *J* = 6.9 Hz, 2 × 3 H, CH*Me*$_2$), 3.05 (sept, *J* = 6.9 Hz, 1 H, C*H*Me$_2$), 3.24 (dd, *J* = 16.2, 7.4 Hz, 1 H, 3-H), 3.34 (dd, *J* = 16.2, 9.1 Hz, 1 H, 3-H), 3.70 (s, 3 H, CO_2Me), 3.77 (ddd, *J* = 9.1, 7.4, 7.1 Hz, 1 H, 2-H), 4.69 (d, *J* = 7.1 Hz, 1 H, 1-H), 7.15-7.23 (m, 4 H, Ar) ppm; the spectrum indicates the presence of ca. 10% of the corresponding *cis*-isomer.

^{13}C NMR ($CDCl_3$, 126 MHz): δ = 17.9, 18.7 (2 q, CH*Me*$_2$), 35.2 (t, C-3), 40.7 (d, C*H*Me$_2$), 46.4 (d, C-2), 58.6 (d, C-1), 123.9, 125.0, 127.0, 127.9, 140.0, 142.0 (4 d, 2 s, Ar), 52.2, 174.6 (q, s, CO_2Me), 212.9 (s, C=O) ppm.

HRMS (ESI-TOF): $C_{15}H_{18}O_3$ Na^+ calcd: 269.1148; found found 269.1155; $[M + K]^+$ calcd.: 285.0888; found: 285.1061.

Methyl (3a*RS*,4*RS*,9b*SR*)- (11a) and (3a*SR*,4*RS*,9b*SR*)-9b-hydroxy-2,3,3a,4,5,9b-hexahydro-1*H*-cyclo-penta[*a*]naphthalene-4-carboxylates (11b):

According to the **GP1**: compound **3a/3b** (200 mg, 0.54 mmol, dr 2:1), $Pd(PPh_3)_4$ (31 mg, 27 µmol), NEt_3 (190 mg, 1.88 mmol), DMF (3.0 mL), 110 °C, 3 d. Column chromatography (silica gel, hexanes/ethyl acetate 85:15) provided 20 mg (15%) of **11a** and 26 mg (20%) of **11b** and as colorless oils.

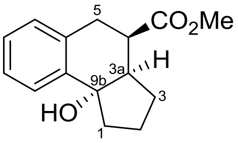

11a

^{1}H NMR ($CDCl_3$, 700 MHz): δ = 1.51 (dtd, *J* = 13.4, 7.7, 5.7 Hz, 1 H, 3-H), 1.67-1.72, 1.73-1.78, 1.86-1.92, 2.17-2.23 (4 m, 3 x 1 H, 2 H, 1-H, 2-H, 3-H), 2.43 (s, 1 H, OH), 2.51 (td, *J* ≈ 9.0, 5.7 Hz, 1 H, 3a-H), 2.65 (td, *J* ≈ 9.0, 4.6 Hz, 1 H, 4-H), 2.94 (dd, *J* = 15.9, 4.6 Hz, 1 H, 5-H), 3.02 (dd, *J* = 15.9, 9.0 Hz, 1 H, 5-H), 3.67 (s, 3 H, CO_2Me), 7.05 (dd, *J* = 7.5, 0.7 Hz, 1 H, Ar), 7.16 (td, *J* = 7.5, 1.3 Hz, 1 H, Ar), 7.25 (td, *J* = 7.6, 1.3 Hz, 1 H, Ar), 7.56 (dd, *J* = 7.6, 0.7 Hz, 1 H, Ar) ppm.

^{13}C NMR ($CDCl_3$, 176 MHz): δ = 22.7, 30.0, 31.5, 42.3 (4 t, C-2, C-3, C-5, C-1), 44.8 (d, C-4), 49.2 (d, C-3a), 80.7 (s, C-9b), 127.0, 127.16, 127.20, 127.9, 133.1, 142.1 (4 d, 2 s, Ar), 51.9, 175.6 (q, s, CO_2Me) ppm.

IR (neat): $\tilde{\nu}$ = 3415 (br, O-H), 3060-2850 (=C-H, C-H), 1730 (C=O) cm^{-1}.

MS (EI = 70 eV): *m/z* (%) = 246 (4) $[M]^+$, 187 (44), 169 (100), 145 (98), 115 (49).

HRMS (80 eV): calcd. for $C_{15}H_{18}O_3$ $[M]^+$ = 246.1256; found: 246.1245.

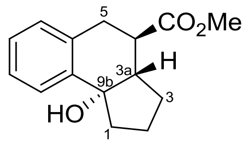

11b

^{1}H NMR ($CDCl_3$, 700 MHz): δ = 1.34-1.40, 1.45-1.51 (2 m, 2 x 1 H, 2-H, 3-H), 1.73 (dddd, *J* ≈ 15.7, 12.7, 7.7, 6.7 Hz, 1 H, 2-H), 1.81 (dtd, *J* ≈ 12.7, 8.5, 4.1 Hz, 1 H, 3-H), 2.04 (dt, *J* = 13.1, 6.7 Hz, 1 H, 1-H), 2.12 (dt, *J* = 13.1, 7.7 Hz, 1 H, 1-H), 2.70 (dt, *J* = 9.8, 4.1 Hz, 1 H, 3a-H), 2.88 (d, *J* = 13.0 Hz, 1 H, 5-H), 2.95-3.02 (m, 2 H, 4-H, 5-H), 3.72 (s, 3 H, CO_2Me), 7.14 (d, *J* = 7.6 Hz, 1 H, Ar), 7.20 (td, *J* = 7.4, 1.2 Hz, 1 H, Ar), 7.27 (t, *J* = 7.4 Hz, 1 H, Ar), 7.57 (d, *J* = 7.6 Hz, 1 H, Ar) ppm; the signal of the OH could not be unambiguously assigned.

^{1}H NMR ($CDCl_3$, 500 MHz): δ = 1.32-1.40, 1.43-1.51, 1.68-1.84, 2.01-2.14 (4 m, 2 × 1 H, 2 × 2 H, 1-H, 2-H, 3-H), 2.68 (dt, *J* = 3.4, 9.6 Hz, 1 H, 3a-H), 2.83-3.02 (m, 3 H, 4-H, 5-H), 3.72 (s, 3 H, CO_2Me), 7.12-7.14, 7.18-7.21, 7.25-7.28, 7.56-7.58 (4 m, 4 × 1 H, Ar) ppm.

^{13}C NMR ($CDCl_3$, 176 MHz): δ = 21.9 (t, C-2), 24.7 (t, C-3), 26.8 (t, C-5), 39.1 (d, C-4), 42.6 (t, C-1), 48.1 (d, C-3a), 80.3 (s, C-9b), 126.9, 127.2, 127.4, 128.6, 134.8, 140.9 (4 d, 2 s, Ar), 51.7, 175.0 (q, s, CO_2Me) ppm.

IR (neat): $\tilde{\nu}$ = 3415 (br, O-H), 3060-2870 (=C-H, C-H), 1730 (C=O) cm^{-1}.

MS (EI = 70 eV): *m/z* (%) = 246 (16) $[M]^+$, 187 (86), 169 (17), 145 (100), 115 (40).

HRMS (80 eV): $C_{15}H_{18}O_3^+$ calcd.: 246.1256; found: 246.1250.

Methyl (3a*RS*,4*RS*,9b*SR*)-9b-(4-nitrobenzoyloxy)-2,3,3a,4,5,9b-hexahydro-1*H*-cyclopenta[a]naphthalene-4-carboxylate (12a):

According to **GP2**: compound **11a** (25 mg, 0.10 mmol), *p*-nitrobenzoyl chloride (20 mg, 0.11 mmol), NEt_3 (12 mg, 0.12 mmol), DMAP (9 mg, 0.08 mmol) in CH_2Cl_2 (1 mL) provided the crude product. Purification by filtration through a pad of alumina (elution with $CHCl_3$) afforded 29 mg (73%) of **12a** a pale yellow solid.

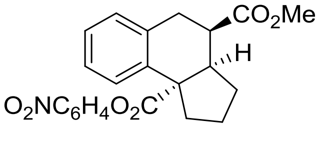

12a

M.p.: 124-126 °C.

^{1}H NMR ($CDCl_3$, 500 MHz): δ = 1.53-1.62, 1.89-2.10, 2.19-2.27 (3 m, 1 H, 4 H, 1 H, 1-H, 2-H, 3-H), 2.61 (td, *J* ≈ 12.0, 3.8 Hz, 1 H, 3a-H), 2.96 (dd, *J* = 15.3, 4.0 Hz, 1 H, 5-H), 3.27 (ddd, *J* ≈ 12.0, 8.0, 4.0 Hz, 1 H, 4-H), 3.38 (dd, *J* ≈ 15.3, 12.0 Hz, 1 H, 5-H), 3.75 (s, 3 H, CO_2Me), 7.14-7.22, 7.27-7.32 (2 m, 3 H, 1 H, Ar), 8.12 (dt, *J* = 9.0, 2.3 Hz, 2 H, Ar), 8.25 (dt, *J* = 9.0, 2.3 Hz, 2 H, Ar) ppm.

^{13}C NMR ($CDCl_3$, 126 MHz): δ = 22.8, 30.0, 33.0, 41.4 (4 t, C-2, C-3, C-5, C-1), 44.3 (d, C-4), 47.8 (d, C-3a), 92.7 (s, C-9b), 123.4, 125.7, 127.0, 127.7, 127.9, 130.5, 136.2, 136.6, 137.2, 150.3 (6 d, 4 s, Ar), 51.9, 174.2 (q, s, CO_2Me), 163.0 (s, CO_2Ar) ppm.

IR (ATR): ṽ = 3080, 3020 (=C-H), 2940, 2850 (C-H), 1730, 1690 (C=O), 1570 (NO_2) cm^{-1}.
HRMS (ESI-TOF): $C_{22}H_{21}NO_6$ Na^+ calcd.: 418.1267; found: 418.1296; $[M+K]^+$ calcd.: 434.1006; found: 434.1048.

Methyl (4b*SR*,8a*RS*,9*RS*)-4b-hydroxy-4b,5,6,7,8,8a,9,10-octahydrophenanthrene-9-carboxylate (13a):
According to the **GP1**: compound **4a** (200 mg, 0.52 mmol), $Pd(PPh_3)_4$ (12 mg, 10 µmol), NEt_3 (160 mg, 1.58 mmol), DMF (2.0 mL), 110 °C, 3 d. Column chromatography (silica gel, hexanes/ethyl acetate 85:15) provided 116 mg (86%) of **13a** as colorless solid.

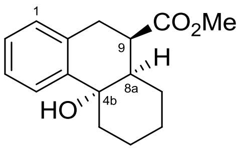

13a

M.p.: 98-99 °C.
^{1}H NMR ($CDCl_3$, 500 MHz): δ = 0.98-1.12, 1.26-1.37, 1.51-1.70 (3 m, 2 H, 1 H, 4 H, 5-H, 6-H, 7-H, 8-H), 1.87 (s, 1 H, OH), 2.28 (ddd, *J* = 12.8, 3.5, 3.4 Hz, 1 H, 8a-H), 2.69-2.73 (m, 1 H, 5-H), 2.95 (dd, *J* = 17.8, 6.1 Hz, 1 H, 10-H), 3.15 (dd, *J* = 17.8, 12.7 Hz, 1 H, 10-H), 3.55 (ddd, *J* = 12.7, 6.1, 3.4 Hz, 1 H, 9-H), 3.74 (s, 3 H, CO_2Me), 7.19-7.25, 7.48-7.50 (2 m, 3 H, 1 H, Ar) ppm.
^{13}C NMR ($CDCl_3$, 126 MHz): δ = 23.8 (t, C-6), 24.1 (t, C-8), 25.5 (t, C-7), 27.3 (t, C-10), 38.2 (d, C-9), 38.3 (t, C-5), 45.1 (d, C-8a), 72.5 (s, C-4b), 125.6, 127.0, 128.0, 130.1, 135.9, 137.1 (4 d, 2 s, Ar), 51.9, 175.6 (q, s, CO_2Me) ppm.
IR (ATR): ṽ = 3460 (br, O-H), 3070-2855 (=C-H, C-H), 1720 (C=O) cm^{-1}.
MS (EI = 70 eV): *m/z* (%) = 260 (27) $[M]^+$, 242 (6), 201 (100), 145 (61).
EA: $C_{16}H_{20}O_3$ (260.3) calcd. (%): C 73.82, H 7.74; found (%): C 73.58, H 7.67.

Methyl (4b*SR*,8a*SR*,9*RS*)-4b-hydroxy-4b,5,6,7,8,8a,9,10-octahydrophenanthrene-9-carboxylate (13b):
According to the **GP1**: compound **4b** (200 mg, 0.52 mmol), $Pd(PPh_3)_4$ (12 mg, 10 µmol), NEt_3 (160 mg, 1.58 mmol), DMF (2.0 mL), 110 °C, 3 d. Column chromatography (silica gel, hexanes/ethyl acetate 85:15) provided 108 mg (80%) of **13b** as colorless oil.

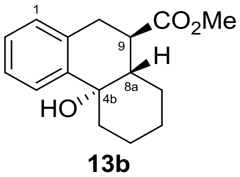

13b

^{1}H NMR ($CDCl_3$, 700 MHz): δ = 1.28-1.35 (m, 1 H, 7-H), 1.42-1.50 (m, 2 H, 5-H, 8-H), 1.60 (qd, *J* ≈ 13.0, 3.5 Hz, 1 H, 8-H), 1.68-1.73 (m, 1 H, 7-H), 1.77-1.81 (m, 2 H, 6-H), 1.82 (ddd, *J* = 12.4, 11.6, 3.5 Hz, 1 H, 8a-H), 2.51-2.54 (m, 1 H, 5-H), 2.91 (td, *J* = 11.6, 5.4 Hz, 1 H, 9-H), 2.98 (dd, *J* = 16.7, 5.4 Hz,

1 H, 10-H), 3.12 (ddd, J = 16.7, 11.6, 0.6 Hz, 1 H, 10-H), 3.71 (s, 3 H, CO_2Me), 7.08-7.12, 7.19-7.23, 7.48-7.52 (3 m, 1 H, 2 H, 1 H, Ar) ppm.

^{13}C NMR ($CDCl_3$, 126 MHz): δ = 21.6, 25.35, 25.41 (3 t, C-6, C-7, C-8), 33.1 (t, C- 10), 36.4 (t, C-5), 41.7 (d, C-9), 45.1 (d, C-8a), 69.6 (s, C-4b), 124.8, 126.5, 127.8, 129.2, 134.5, 140.9 (4 d, 2 s, Ar), 51.5, 176.2 (q, s, CO2Me) ppm.

IR (neat): ṽ = 3500 (O-H), 3060, 3030 (=C-H), 2935, 2850 (C-H), 1720 (C=O) cm-1.

HRMS (ESI-TOF): $C_{16}H_{20}O_3$ Na^+ calcd.: 283.1310; found: 283.1316.

(4b*SR*,8a*RS*,9*RS*)-4b-(4-Nitrobenzoyloxy)-4b,5,6,7,8,8a,9,10-octahydrophenanthrene-9-carboxylate (14a):

According to **GP2**: compound **13a** (42 mg, 0.16 mmol), *p*-nitrobenzoyl chloride (32 mg, 0.17 mmol), NEt_3 (16 mg, 0.16 mmol), DMAP (15 mg, 0.12 mmol) in CH_2Cl_2 (1 mL) provided the crude product. Purification by column chromatography (alumina, hexanes/ethyl acetate 85:15) gave 44 mg (67%) of **14a** a pale yellow solid. Suitable crystals for the X-ray structure determination were grown from Et_2O/hexanes (1:2) at −10 °C.

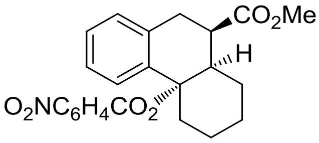

14a

M.p.: 91-93 °C.

^{1}H NMR ($CDCl_3$, 500 MHz): δ = 1.11-1.24, 1.60-1.68, 1.75-1.80 (3 m, 2 x 2 H, 1 H, 6-H, 7-H, 8-H), 1.42 (qt, J ≈ 12.8, 3.5 Hz, 1 H, 7-H), 2.06 (td, J = 13.5, 3.6 Hz, 1 H, 5-H), 2.92 (dt, J = 12.8, 3.5 Hz, 1 H, 8a-H), 3.05 (dd, J = 18.1, 6.5 Hz, 1 H, 10-H), 3.21 (dd, J = 18.1, 12.1 Hz, 1 H, 10-H), 3.59 (ddd, J = 12.1, 6.5, 3.5 Hz, 1 H, 9-H), 3.62-3.67 (m, 1 H, 5-H) 3.81 (s, 3 H, CO_2Me), 7.19-7.24, 7.27-7.32, 7.95-8.01 (3 m, 1 H, 2 H, 1 H, Ar), 8.04 (d, J = 8.8 Hz, 2 H, Ar), 8.21 (d, J = 8.8 Hz, 2 H, Ar) ppm.

^{13}C NMR ($CDCl_3$, 126 MHz): δ = 23.3, 23.8, 25.1, 27.1 (4 t, C-6, C-8, C-7, C-10), 33.4 (t, C-5), 38.2 (d, C-9), 43.3 (d, C-8a), 85.9 (s, C-4b), 123.4, 125.9, 128.9, 129.7, 129.9, 130.5, 131.0, 136.4, 137.2, 150.3 (6 d, 4 s, Ar), 52.0, 174.6 (q, s, CO_2Me), 162.8 (s, CO_2Ar) ppm.

IR (ATR): ṽ = 3110 (=C-H), 2940, 2860 (C-H), 1720 (C=O), 1525 (NO_2) cm^{-1}.

HRMS (ESI-TOF): $C_{23}H_{23}NO_6$ Na^+ calcd.: 432.1423; found 432.1422; $[M+K]^+$ calcd.: 448.1157; found: 448.1155.

EA: $C_{23}H_{23}NO_6$ (409.4) calcd. (%): C 67.47, H 5.66, N 3.42; found (%): C 67.52, H 5.59, N 3.42.

Methyl (6*RS*,6a*RS*,11a*RS*)-11a-hydroxy-6,6a,7,8,9,10,11,11a-octahydro-5*H*-cyclohepta[*a*]naphthalene-6-carboxylate (15a):

According to the **GP1**: compound **5a/5b** (150 mg, 0.37 mmol, d.r. ca. 1:1), $Pd(PPh_3)_4$ (9 mg, 7.8 µmol), NEt_3 (131 mg, 1.30 mmol), DMF (1.5 mL), 120 °C, 1.5 h, microwave (400 Watt). The obtained mixture of compounds was purified by column chromatography (silica gel, hexanes/ethyl acetate 9:1) affording 26

mg (26%) of pure **15a** as pale yellow solid. Suitable crystals for an X-ray crystal structure analysis were obtained by recrystallization from hexanes/diethyl ether.

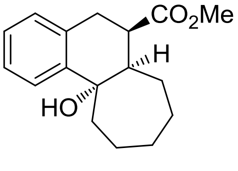

15a

^{1}H NMR ($CDCl_3$, 700 MHz): δ = 1.12 (dddd, *J* = 14.4, 12.6, 9.5, 1.8 Hz, 1 H, 7-H), 1.18-1.26, 1.41-1.49, 1.66-1.72, 1.74-1.81, 1.83-1.89 (5 m, 1 H, 2 H, 1 H, 1 H, 2 H, 7-H, 8-H, 9-H, 10-H), 1.92 (s_{br}, 1 H, OH), 1.93 (ddd, *J* = 14.9, 10.6, 0.8 Hz, 1 H, 11-H), 2.53 (dd, *J* = 9.5, 3.1 Hz, 1 H, 6a-H), 2.59 (dd, *J* = 14.9, 8.8 Hz, 1 H, 11-H), 2.91 (dd, *J* = 16.9, 4.7 Hz, 1 H, 5-H), 3.01 (dd, *J* = 16.9, 13.0 Hz, 1 H, 5-H), 3.24 (ddd, *J* = 13.0, 4.7, 3.1 Hz, 1 H, 6-H), 3.75 (s, 3 H, CO_2Me), 7.15 (d, *J* = 7.7 Hz, 1 H, Ar), 7.21 (td, *J* = 7.4, 1.3 Hz, 1 H, Ar), 7.24 (t, *J* = 7.4 Hz, 1 H, Ar), 7.50 (dd, *J* = 7.7, 1.3 Hz, 1 H, Ar) ppm.
^{13}C NMR ($CDCl_3$, 176 MHz): δ = 21.7, 24.7, 27.0, 29.8, 30.1, 42.0 (6 t, C-10, C-7, C-5, C-9, C-8, C-11), 41.0 (d, C-6), 47.1 (d, C-6a), 75.3 (s, C-11a), 126.7, 127.1, 127.5, 129.3, 135.0, 140.7 (4 d, 2 s, Ar), 51.7, 175.4 (q, s, CO_2Me) ppm.
IR (ATR): $\tilde{\nu}$ = 3470 (O-H), 3020 (=C-H), 2920, 2850 (C-H), 1710 (C=O) cm^{-1}.
HRMS (ESI-TOF): $C_{17}H_{22}O_3$ Na^+ calcd.: 297.1467; found: 297.1466; $[2M+Na]^+$ cacld.: 517.3036; found: 517.3043.

Methyl (6*RS*,6a*SR*,11a*SR*)-11a-hydroxy-6,6a,7,8,9,10,11,11a-octahydro-5*H*-cyclohepta[*a*]naphthalene-6-carboxylate (15b):

According to the **GP1**: compound **5b** (150 mg, 0.37 mmol, diastereomeric purity 92%), $Pd(PPh_3)_4$ (9 mg, 7.8 µmol), NEt_3 (131 mg, 1.30 mmol), DMF (1.5 mL), 120 °C, 1.5 h, microwave (400 Watt). Column chromatography (silica gel, hexanes/ethyl acetate 85:15) provided 46 mg (45%) of **13b** as colorless solid. Suitable crystals for an X-ray crystal structure analysis were obtained by recrystallization from hexanes/diethyl ether (2:1).

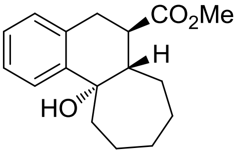

15b

M.p.: 114-117 °C.
^{1}H NMR ($CDCl_3$, 700 MHz): δ = 1.38 (dtd, *J* = 13.8, 5.6, 2.2 Hz, 1 H, 7-H), 1.56, 1.60-1.68, 1.69-1.75, 1.76-1.82 (m_c, 3 m, 1 H, 2 H, 3 H, 1 H, 7-H, 8-H, 9-H, 10-H), 1.95 (dtd, *J* = 15.0, 9.8, 5.5 Hz, 1 H, 11-H), 2.07 (m, 1 H, 6a-H), 2.42 (ddd, *J* = 14.3, 6.0, 2.7 Hz, 1 H, 11-H), 2.68 (ddd, *J* = 11.3, 10.4, 6.0 Hz, 1 H, 6-H), 2.94-3.01 (m, 2 H, 5-H), 3.73 (s, 3 H, CO_2Me), 7.06 (d, *J* = 7.5 Hz, 1 H, Ar), 7.18 (t, *J* = 7.3 Hz, 1 H, Ar), 7.23 (t, *J* = 7.5 Hz, 1 H, Ar), 7.56 (d, *J* = 7.9 Hz, 1 H, Ar) ppm; the signal of the OH could not be unambiguously assigned.

^{13}C NMR ($CDCl_3$, 176 MHz): δ = 23.7, 26.5, 26.6, 27.0, 34.5, 43.9 (6 t, C-8, C-7, C-10, C-9, C-5, C-11), 44.3 (d, C-6), 46.4 (d, C-6a), 73.9 (s, C-11a), 126.7, 126.9, 127.5, 128.6, 134.5, 143.2 (4 d, 2 s, Ar), 51.7, 176.6 (q, s, CO_2Me) ppm.
IR (ATR): ṽ = 3510 (O-H), 3025 (=C-H), 2925–2855 (C-H), 1715 (C=O) cm^{-1}.
HRMS (ESI-TOF): $C_{17}H_{22}O_3$ Na^+ calcd.: 297.1461; found: 297.1475; $[2M+Na]^+$: 571.3030; found: 571.3048.

Methyl 2-methoxy-5,6,7,8,9,10-hexahydrophenanthrene-9-carboxylate (16):
According to the **GP1**: compound **6a/b** (208 mg, 0.50 mmol), $Pd(PPh_3)_4$ (12 mg, 10 µmol), NEt_3 (177 mg, 1.75 mmol), DMF (2 mL), 110 °C, 3 d. Column chromatography (silica gel, hexanes/ethyl acetate 85:15), then HPLC (hexanes/ethyl acetate 19:1) provided 35 mg (24%) of **16** as colorless solid.

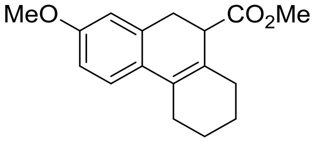

16

M.p.: 58-61 °C.
^{1}H NMR ($CDCl_3$, 500 MHz): δ = 1.62-1.75, 1.77-1.85, 2.10-2.18, 2.27-2.38, 2.44-2.52 (5 m, 3 H, 1 H, 1 H, 2 H, 1 H, 5-H, 6-H, 7-H, 8-H), 2.97 (dd, *J* = 13.4, 5.1 Hz, 1 H, 10-H), 3.06-3.13 (m, 2 H, 9-H, 10-H), 3.62 (s, 3 H, OMe), 3.79 (s, 3 H, CO_2Me), 6.68-6.72 (m, 2 H, Ar), 7.12 (d, *J* = 8.8 Hz, 1 H, Ar) ppm.
^{13}C NMR ($CDCl_3$, 126 MHz): δ = 22.78, 22.79, 25.7, 29.6, 31.5 (5 t, C-5, C-6, C-7, C-8, C-10), 44.8 (d, C-9), 55.1 (q, OMe), 111.1, 113.4, 122.9 (3 d, Ar), 127.8, 128.6, 128.9, 134.9 (4 s, Ar, C-4b, C-8a), 158.1 (s, C-OMe), 51.7, 174.2 (q, s, CO_2Me) ppm.
IR (ATR): ṽ = 3010 (=C-H), 2950-2840 (C-H), 1730 (C=O), 1605 (C=C) cm^{-1}.
HRMS (ESI-TOF): $C_{17}H_{20}O_3$ Na^+ calcd.: 295.1310; found: 295.1313; $[2M+Na]^+$ calcd.: 567.2723; found: 567.2728.

3) References

1. Reissig, H.-U.; Khan, F. A.; Czerwonka, R.; Dinesh, C. U.; Shaikh, A. L.; Zimmer, R. *Eur. J. Org. Chem.* **2006**, 4419-4428.
doi:10.1002/ejoc.200600360
2. Nandanan, E.; Dinesh, C. U.; Reissig, H.-U. *Tetrahedron* **2000**, *56*, 4267-4277.
doi:10.1016/S0040-4020(00)00353-7
3. Saadi, J.; Brüdgam, I.; Reissig, H.-U. *Beilstein J. Org. Chem.* **2010**, *6*, 1229–1245.
doi:10.3762/bjoc.6.141

4) Copies of NMR spectra

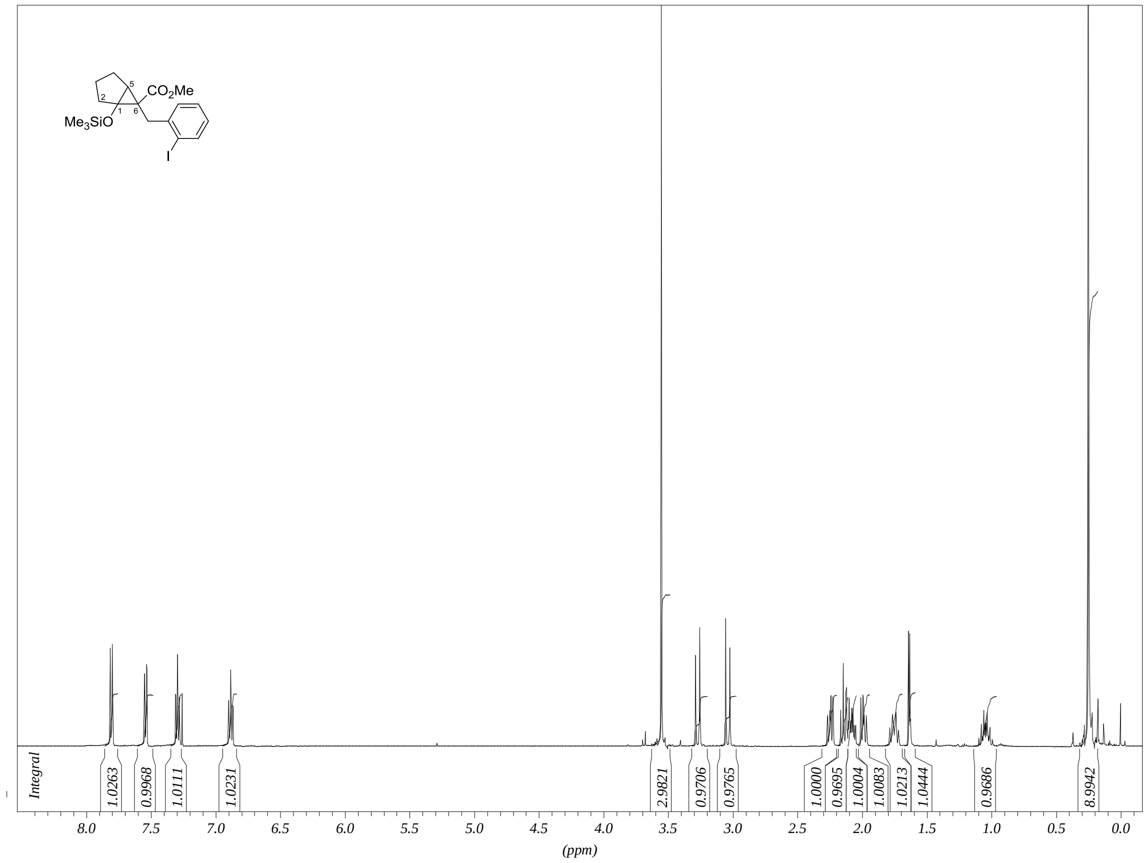

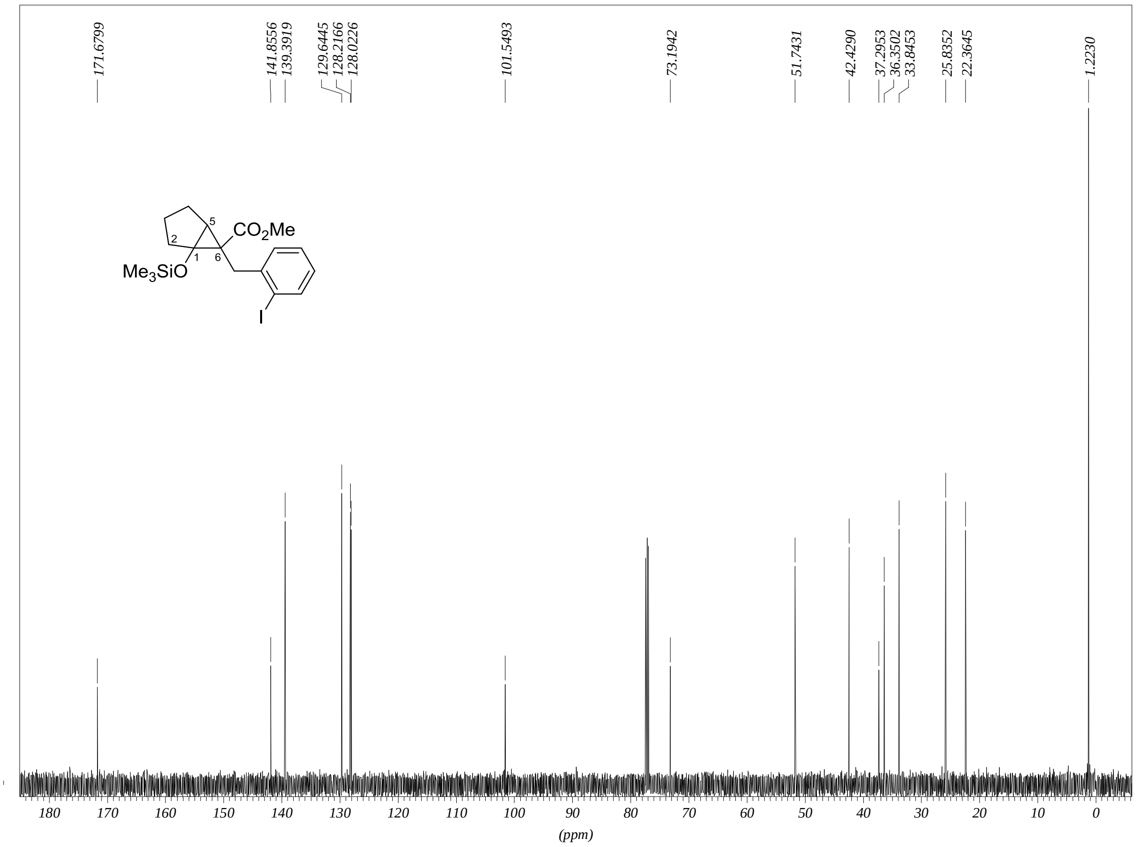

500 MHz, CHLOROFORM-D

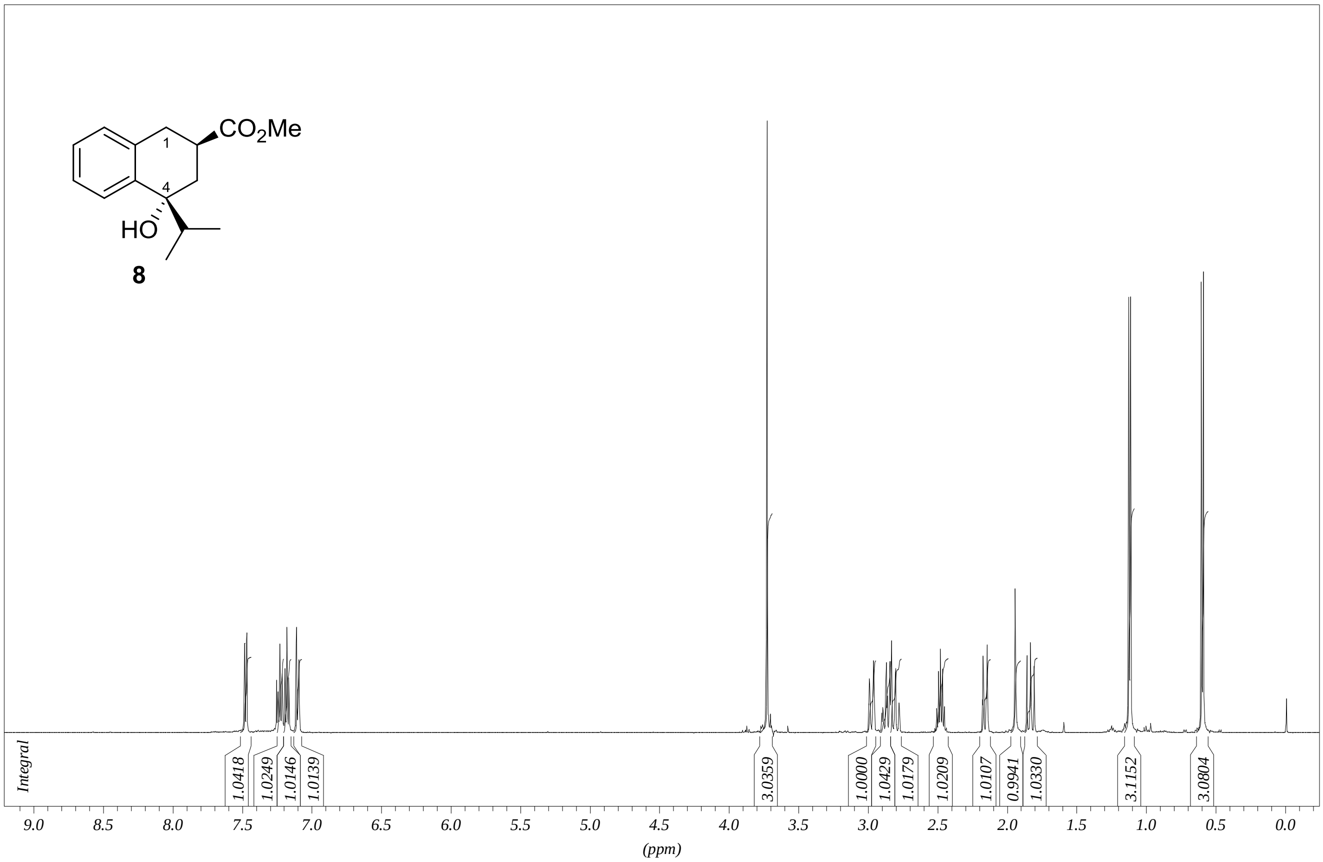

125 MHz, CHLOROFORM-D

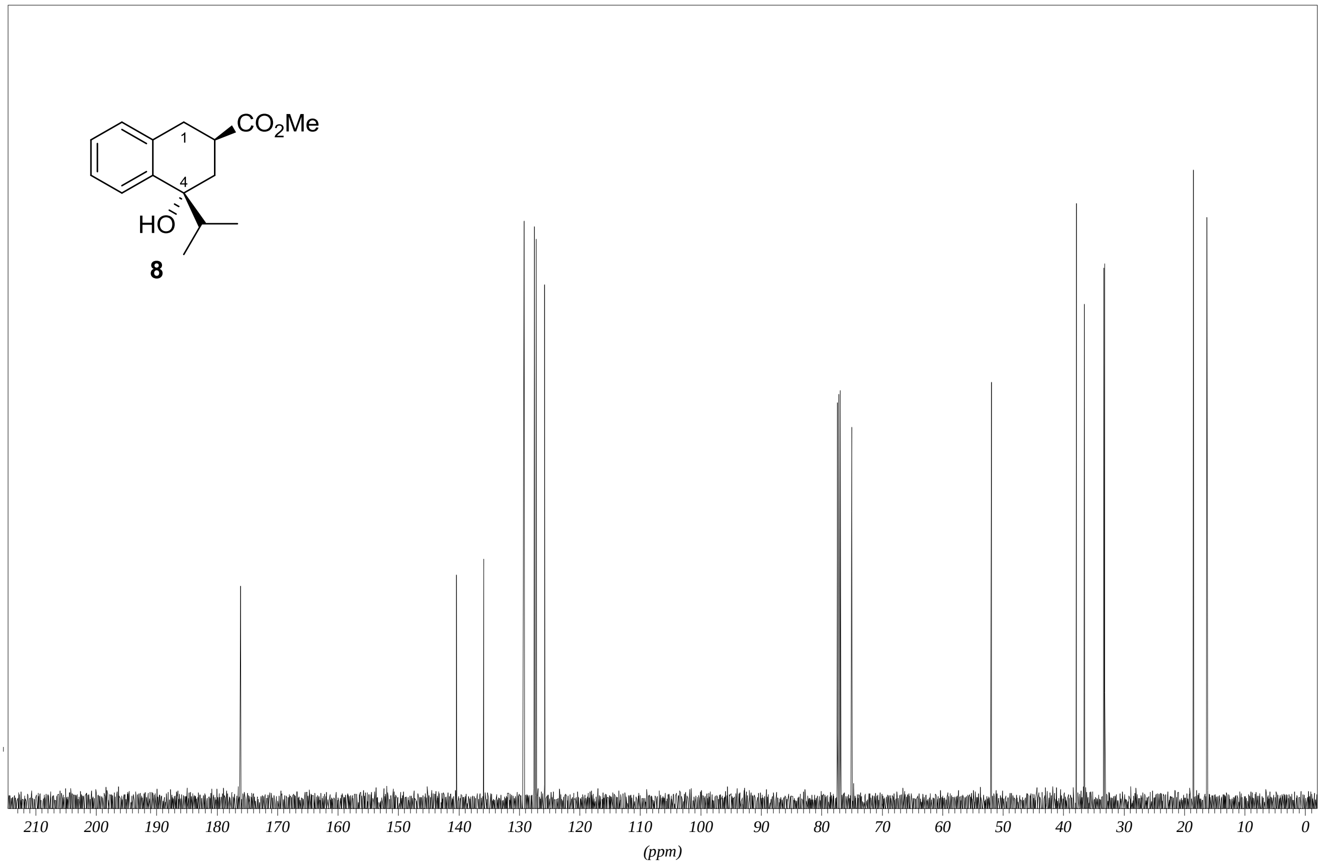

500 MHz, CHLOROFORM-D

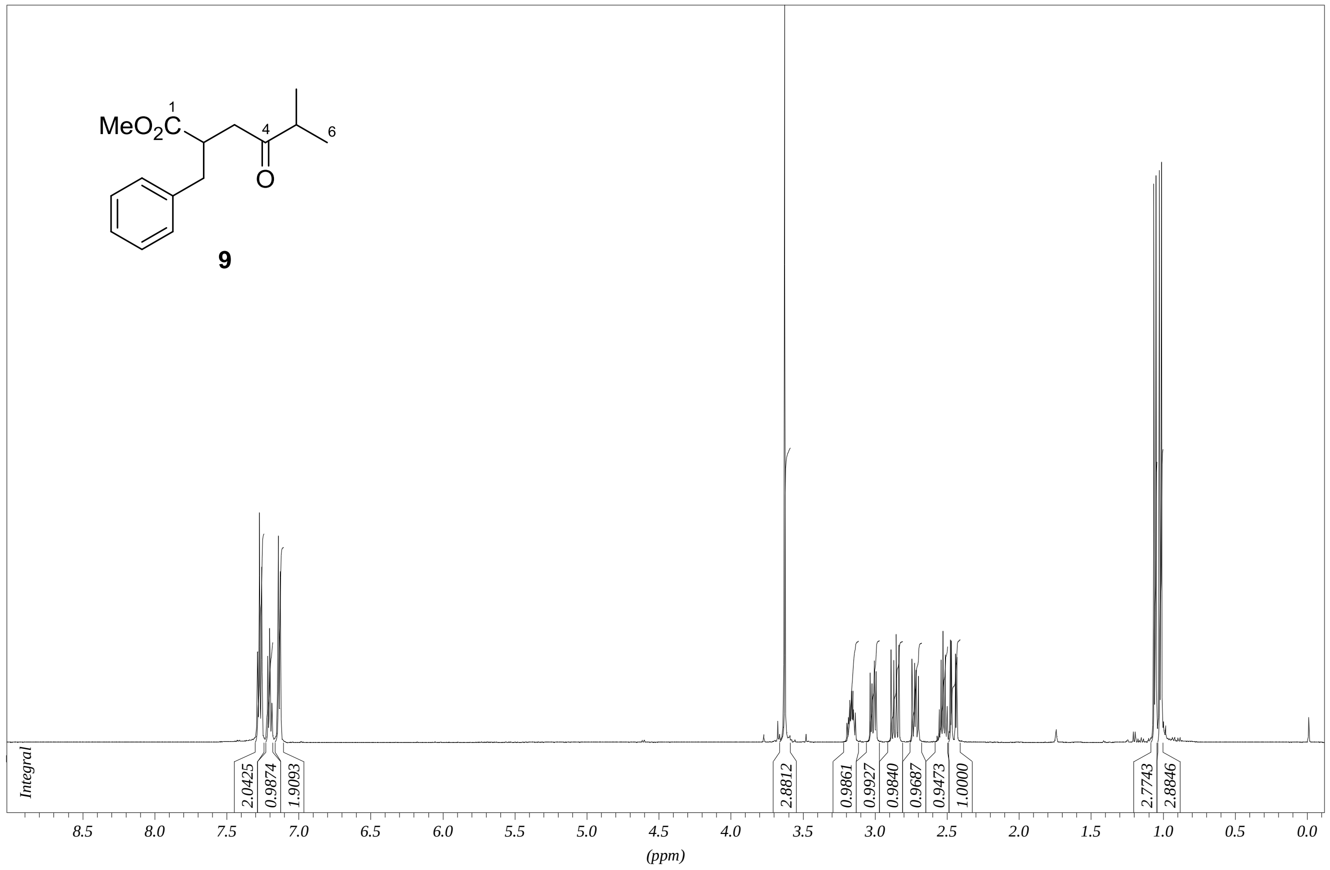

125 MHz, CHLOROFORM-D

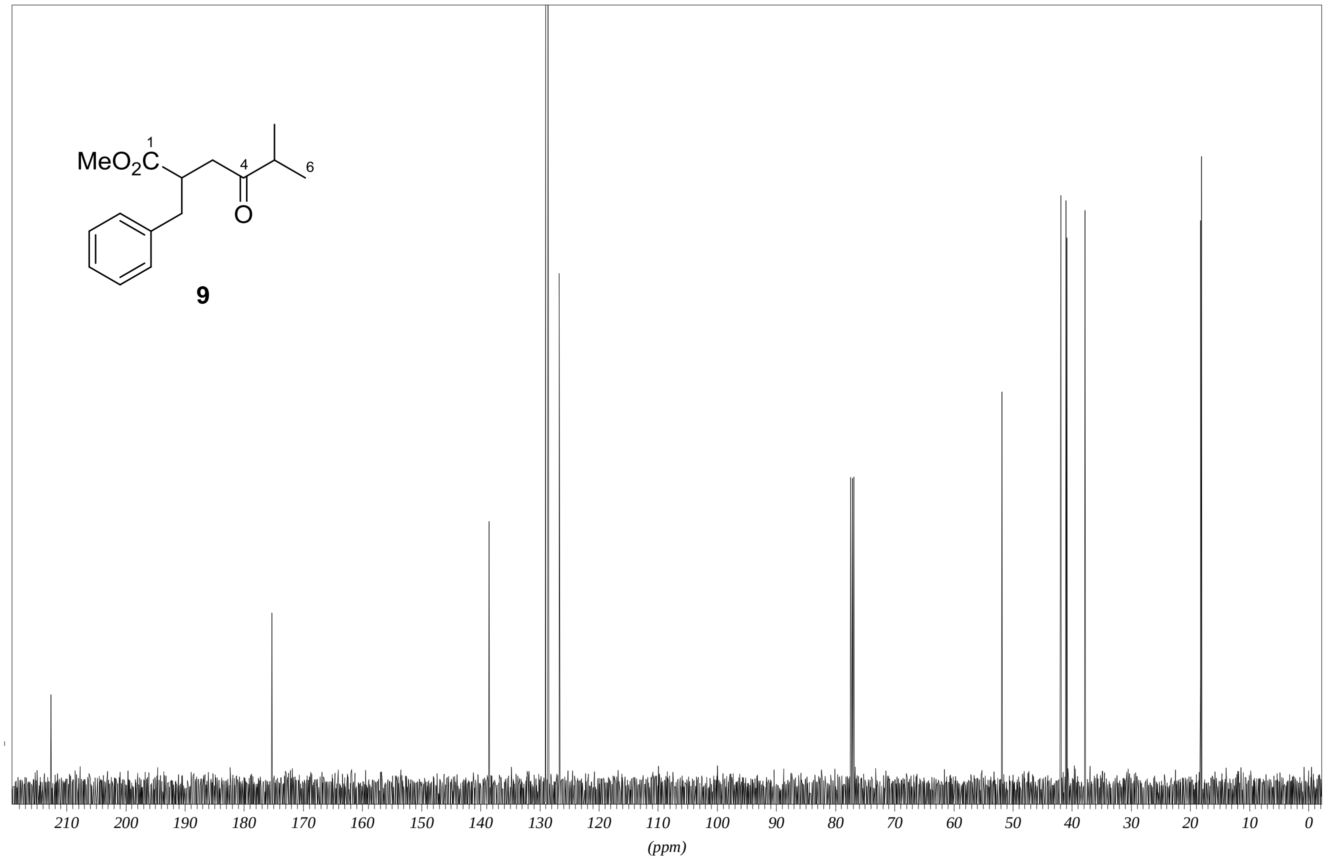

500 MHz, CHLOROFORM-D

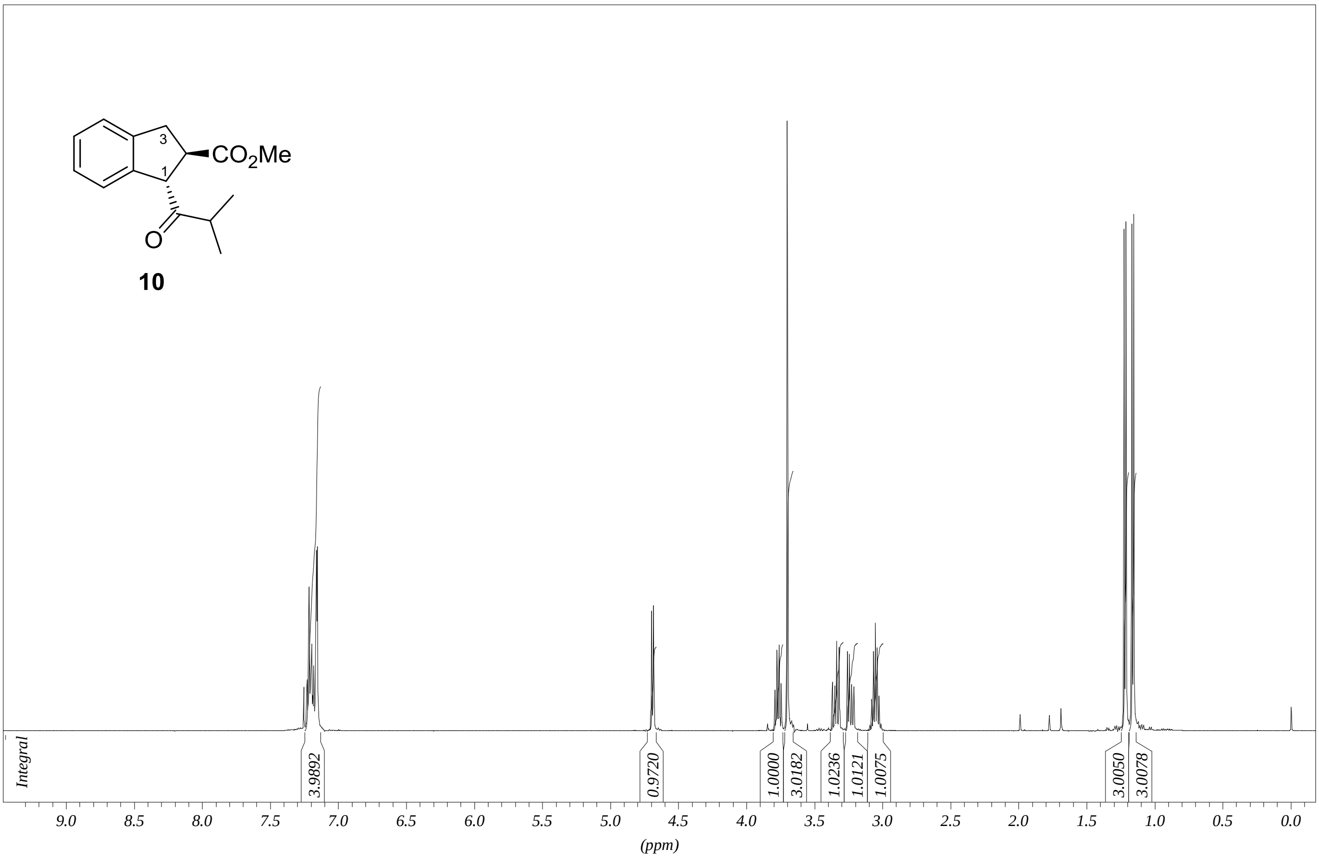

125 MHz, CHLOROFORM-D

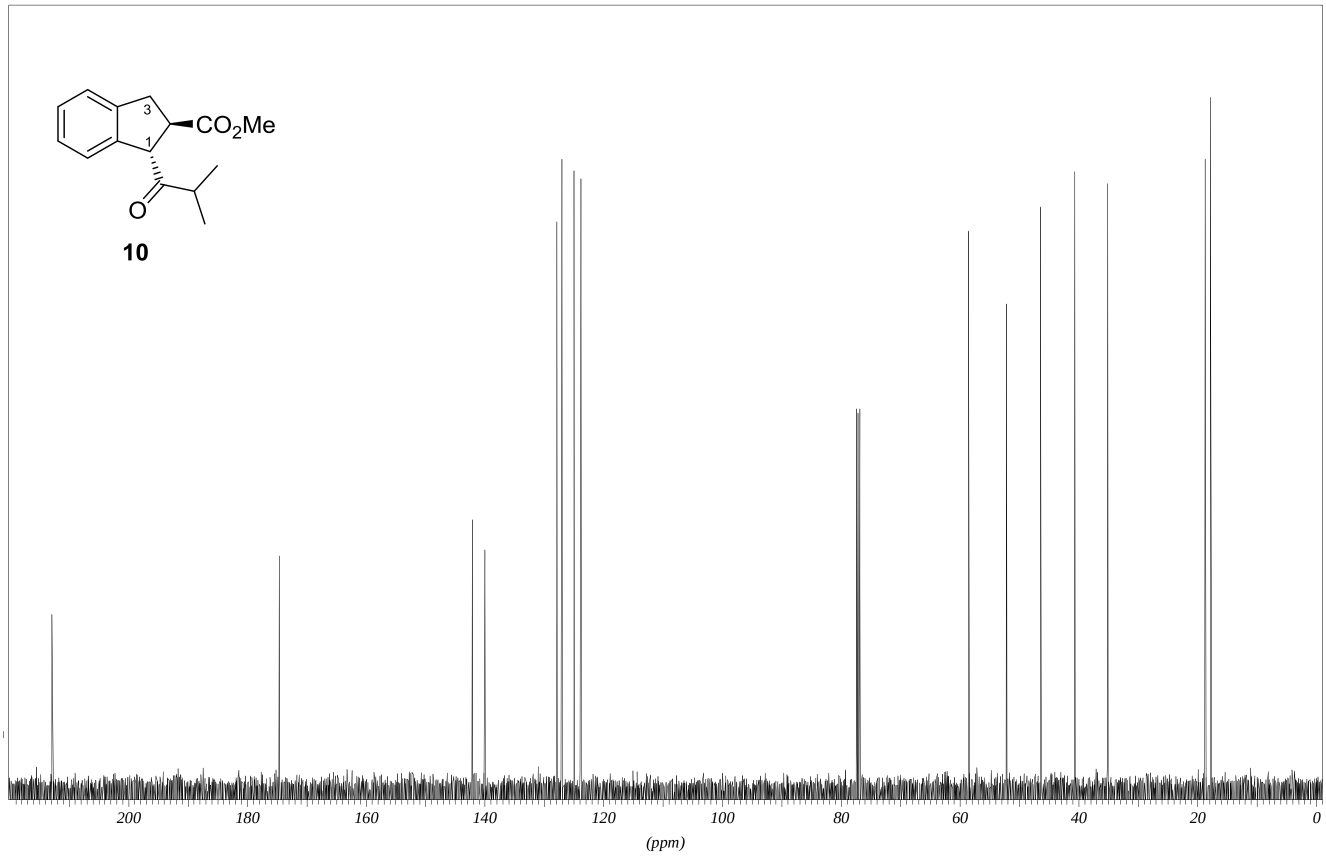

500 MHz, CHLOROFORM-D

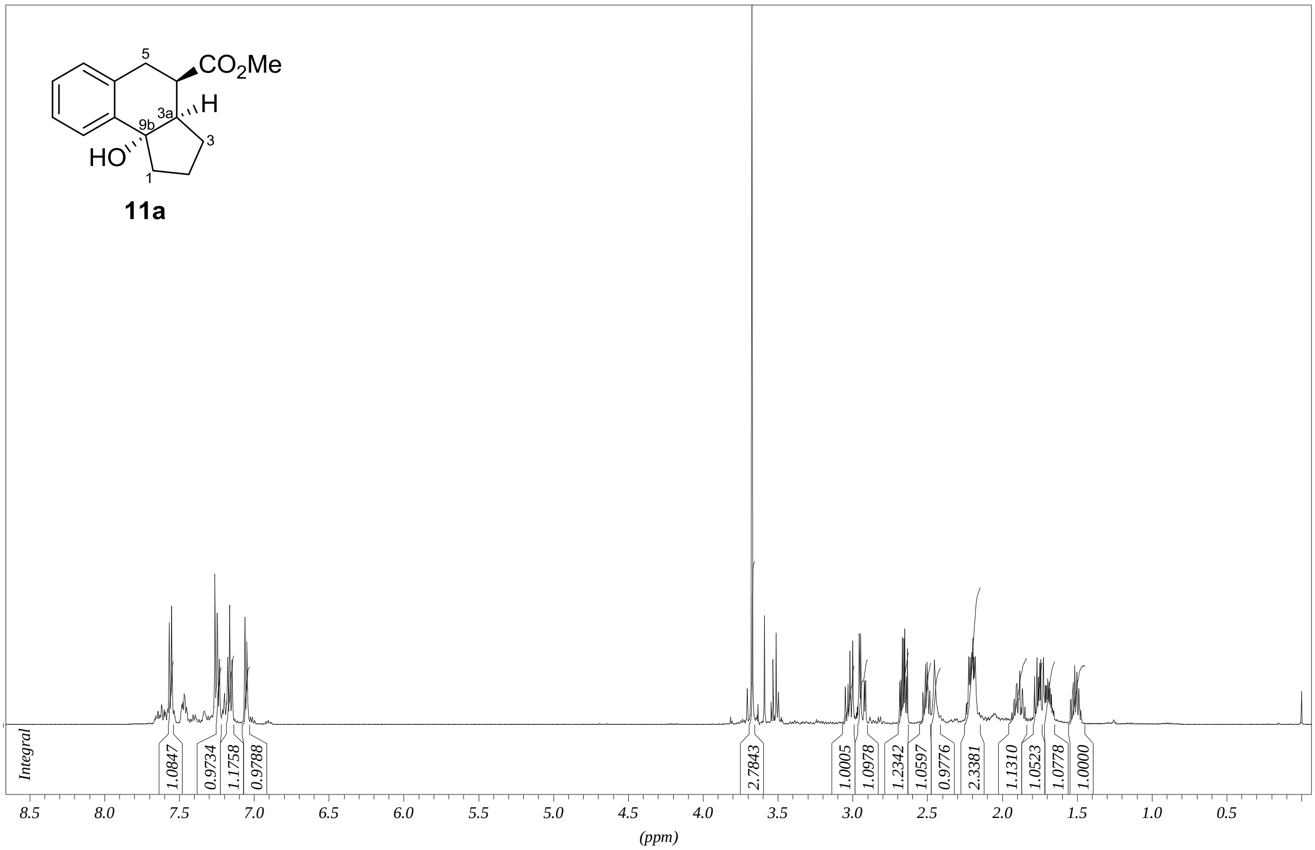

125 MHz, CHLOROFORM-D

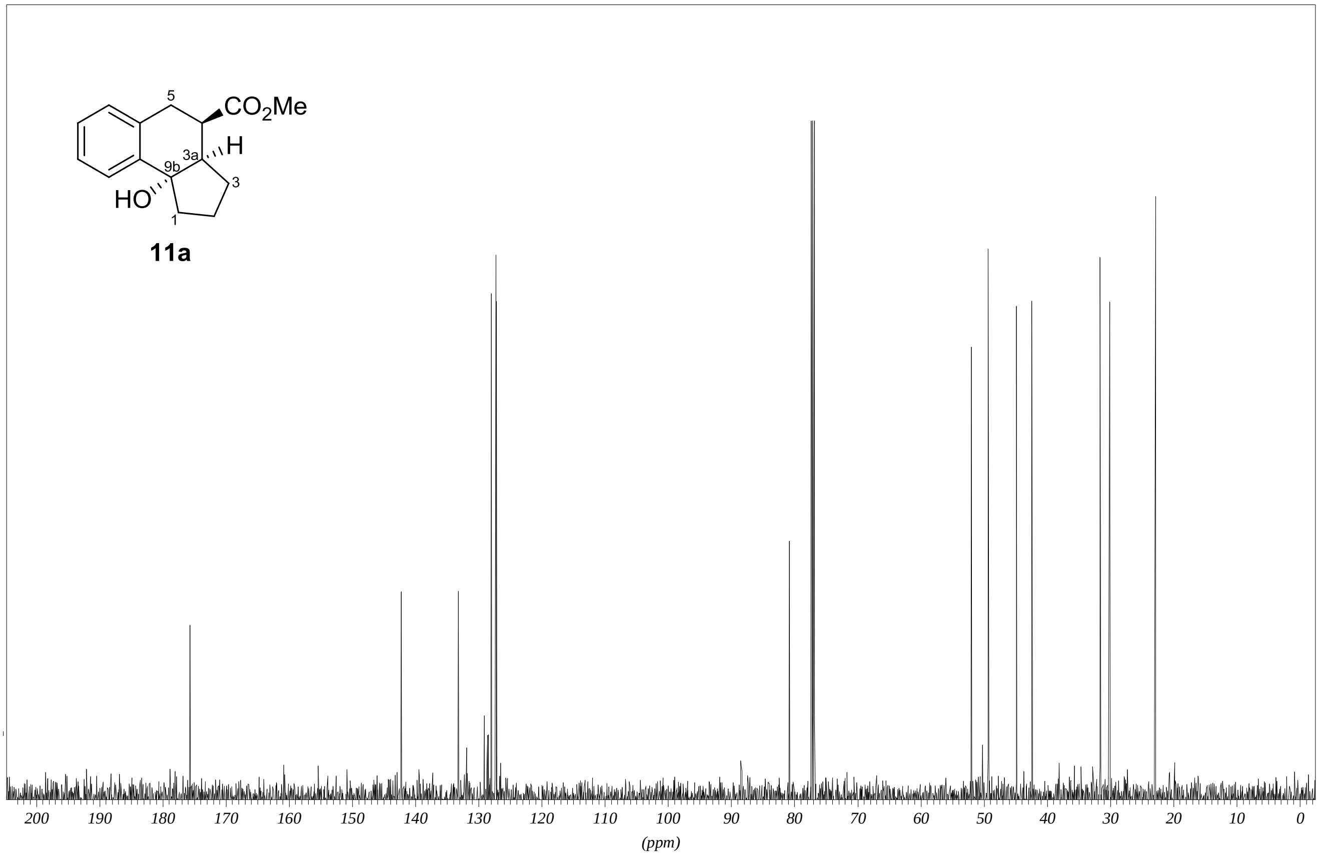

500 MHz, CHLOROFORM-D

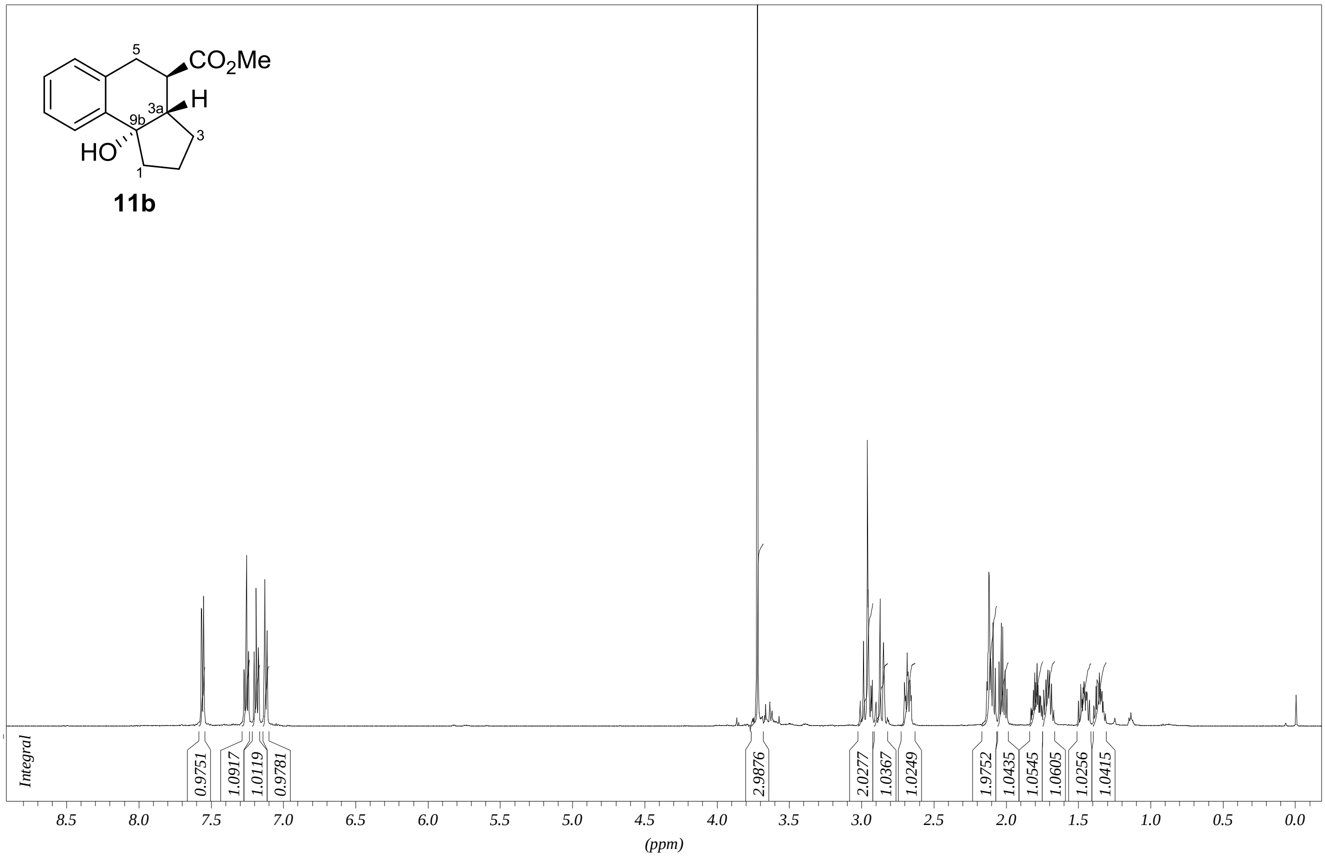

125 MHz, CHLOROFORM-D

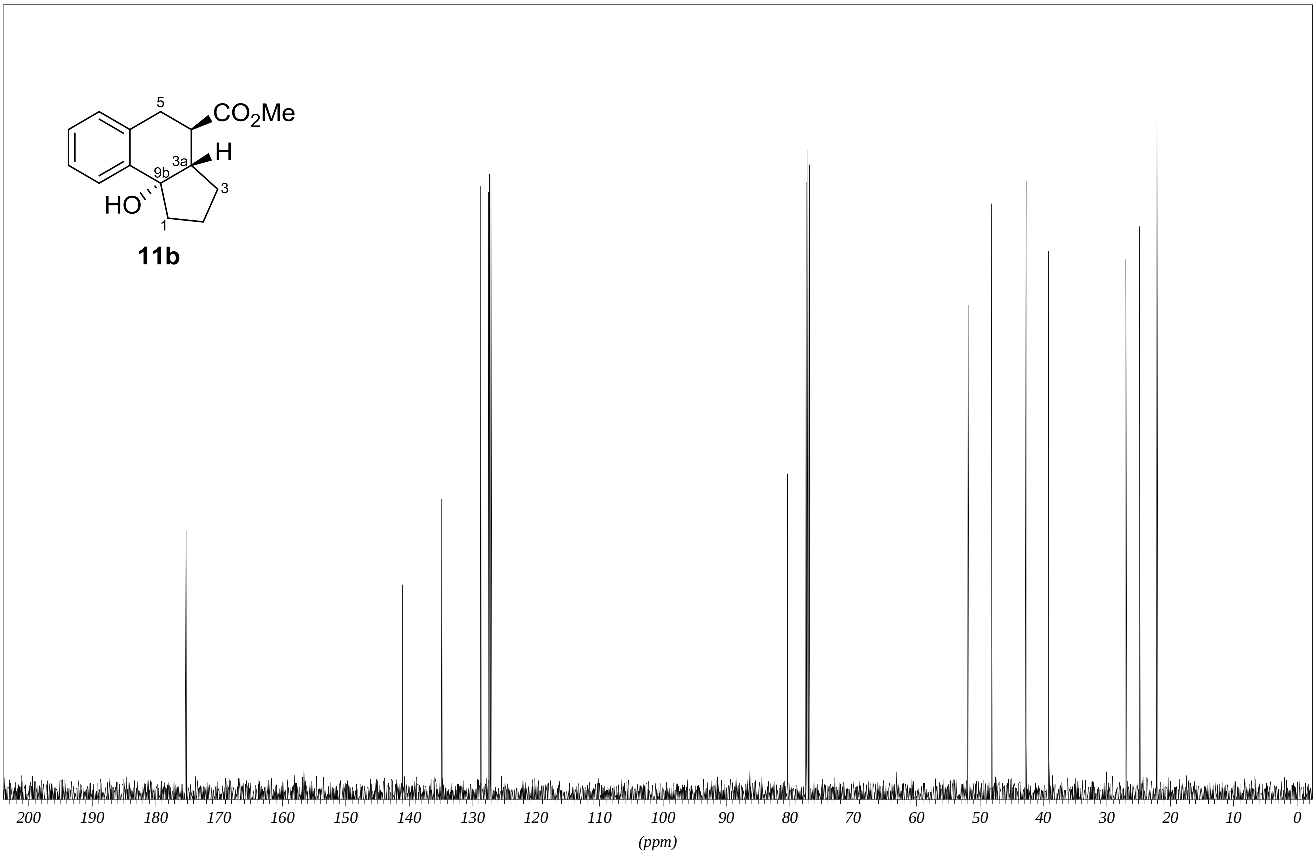

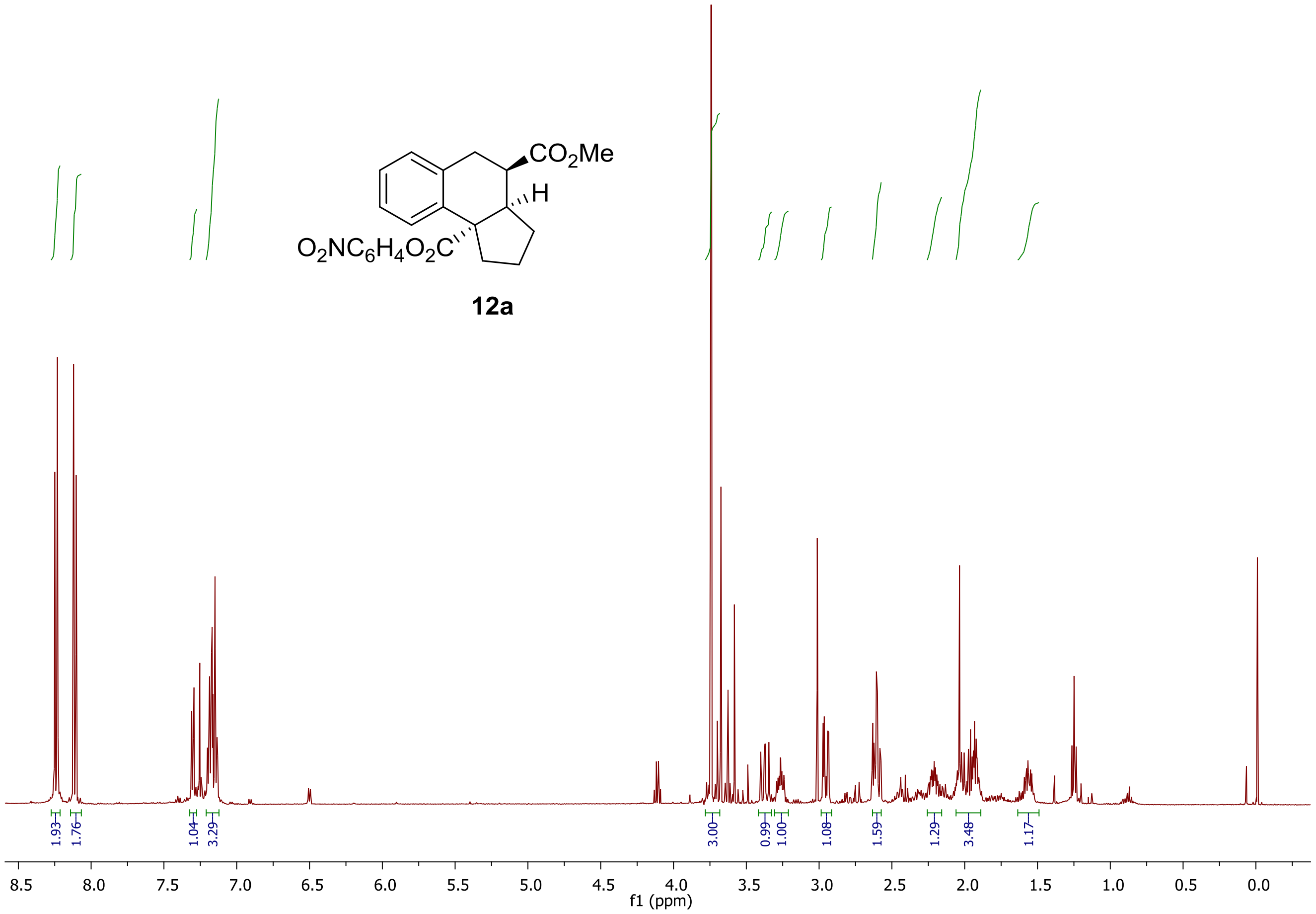

CO2Me
H
O2NC6H4O2C
12a
1.93
1.76
1.04
3.29
3.00
0.99
1.00
1.08
1.59
1.29
3.48
1.17
8.5
8.0
7.5
7.0
6.5
6.0
5.5
5.0
4.5
4.0
3.5
3.0
2.5
2.0
1.5
1.0
0.5
0.0
f1 (ppm)

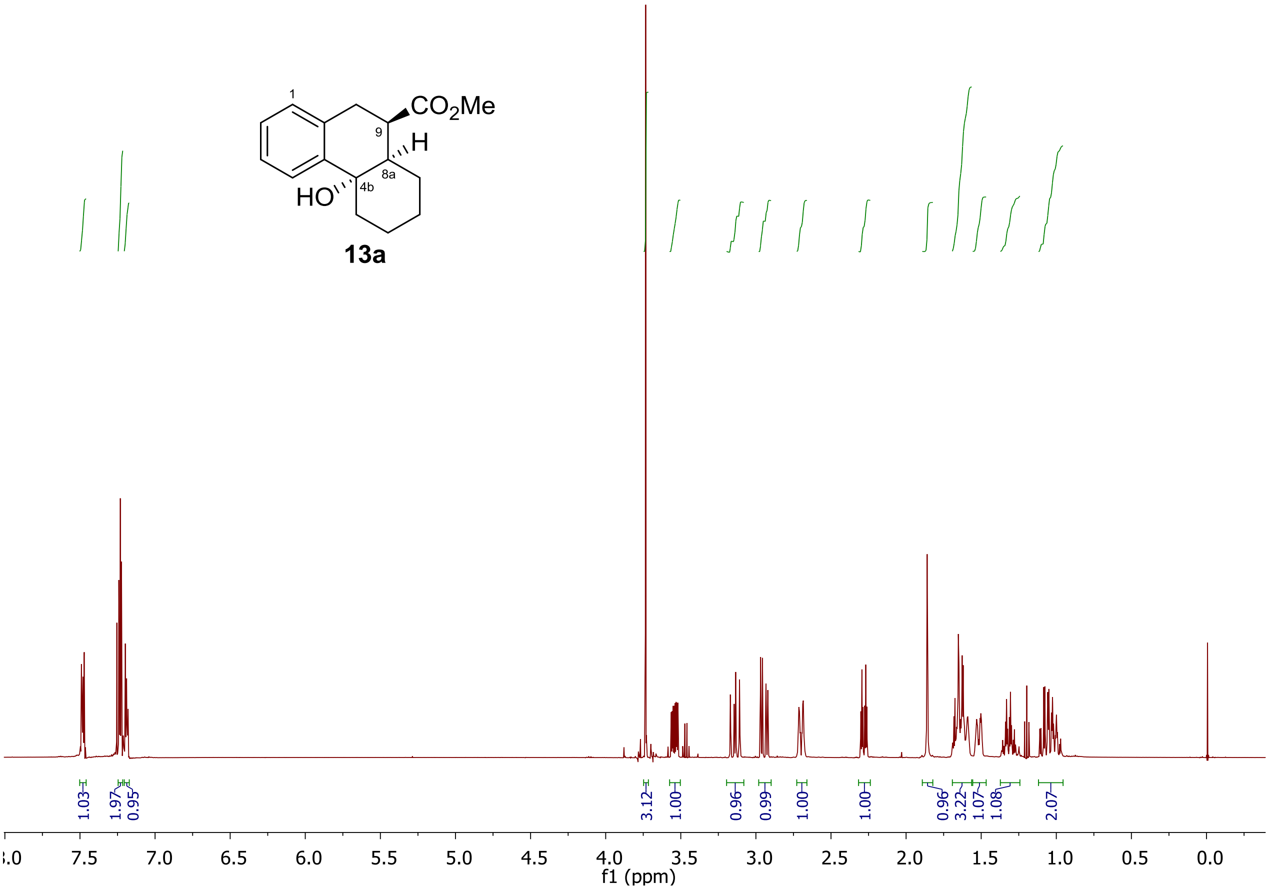
1
CO2Me
9
H
8a
4b
HO
13a
1.03
1.97
0.95
3.12
1.00
0.96
0.99
1.00
1.00
0.96
3.22
1.07
1.08
2.07
7.5
7.0
6.5
6.0
5.5
5.0
4.5
4.0
3.5
3.0
2.5
2.0
1.5
1.0
0.5
0.0
f1 (ppm)

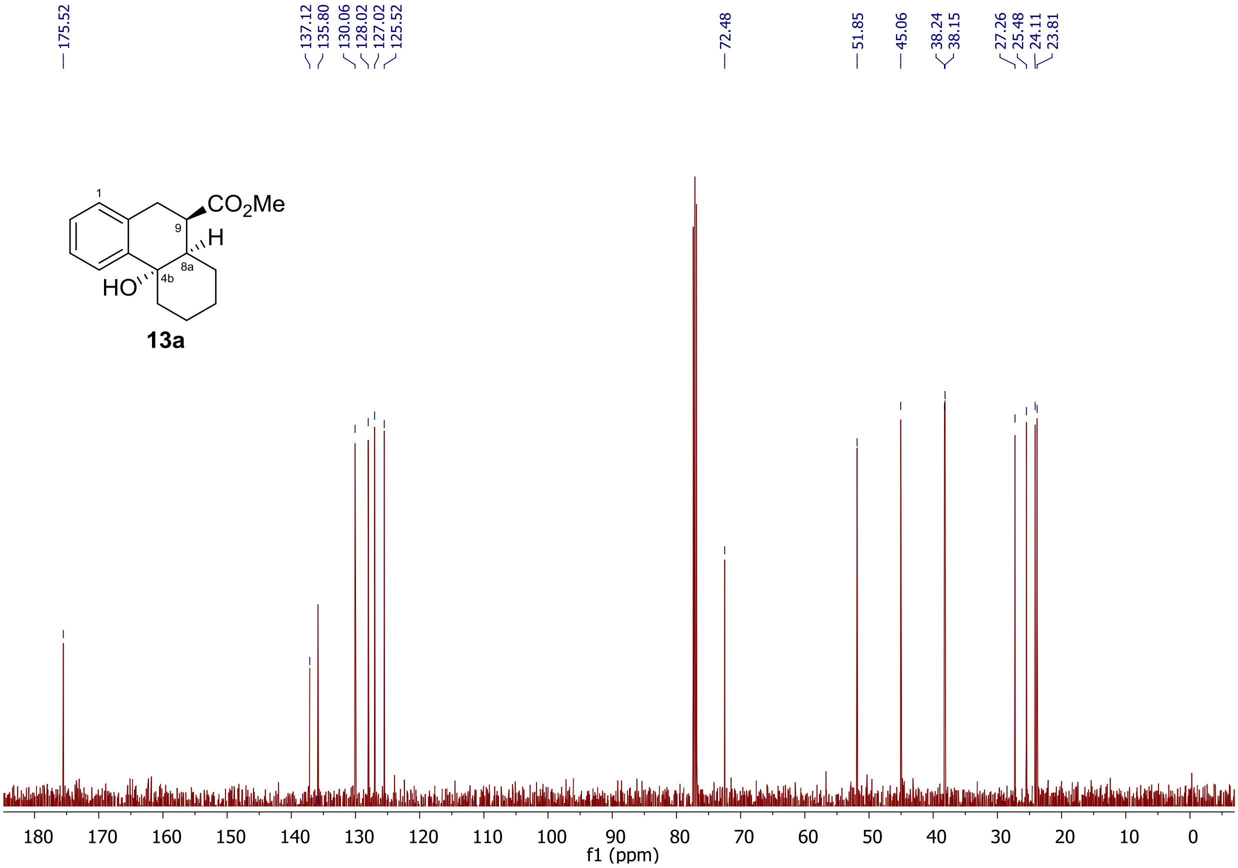
175.52
137.12
135.80
130.06
128.02
127.02
125.52
72.48
51.85
45.06
38.24
38.15
27.26
25.48
24.11
23.81
1
CO2Me
9
H
8a
4b
HO
13a
180
170
160
150
140
130
120
110
100
90
80
70
60
50
40
30
20
10
0
f1 (ppm)

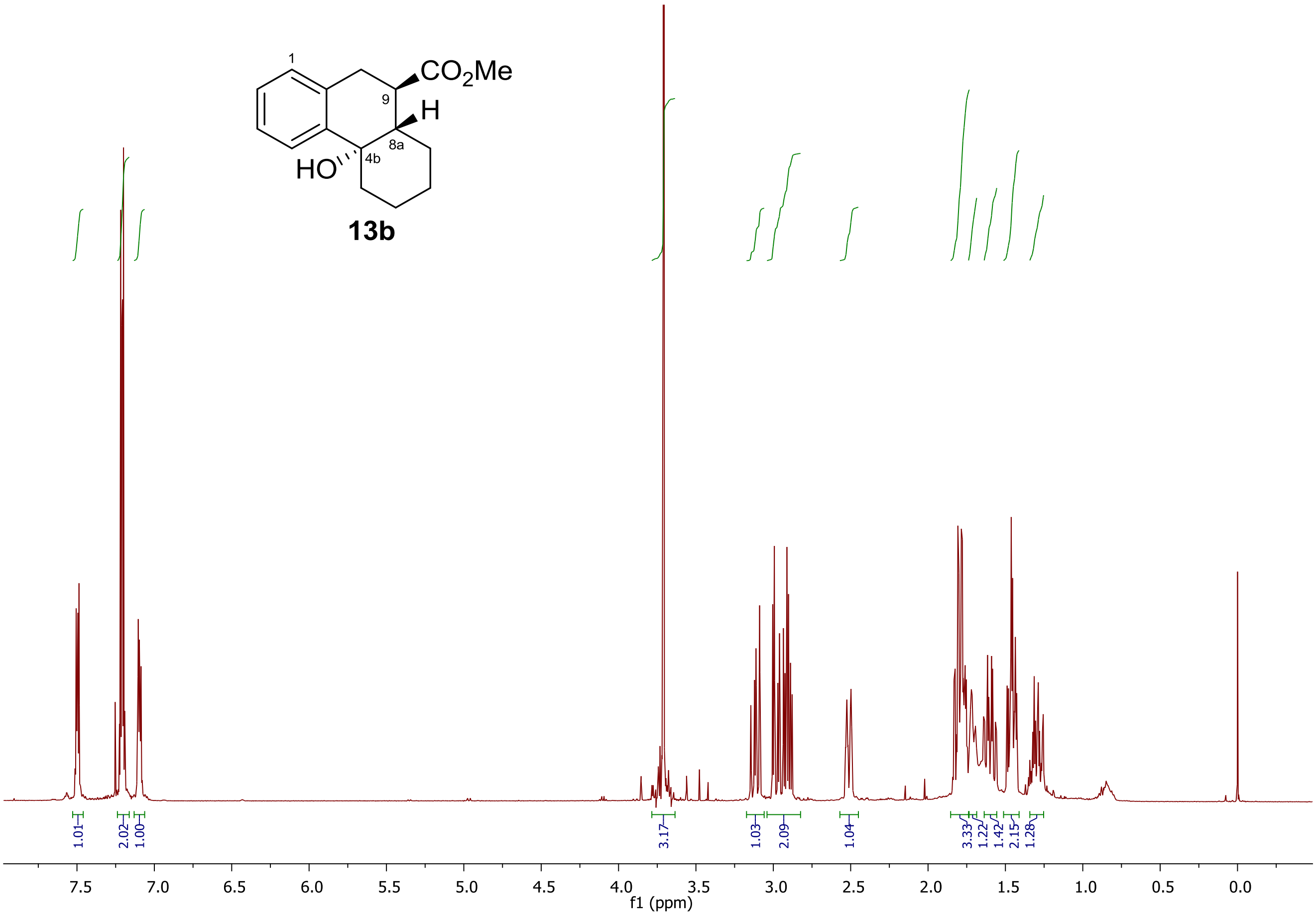

1
9
CO2Me
H
8a
4b
HO
13b
1.01
2.02
1.00
3.17
1.03
2.09
1.04
3.33
1.22
1.42
2.15
1.28
7.5
7.0
6.5
6.0
5.5
5.0
4.5
4.0
3.5
3.0
2.5
2.0
1.5
1.0
0.5
0.0
f1 (ppm)

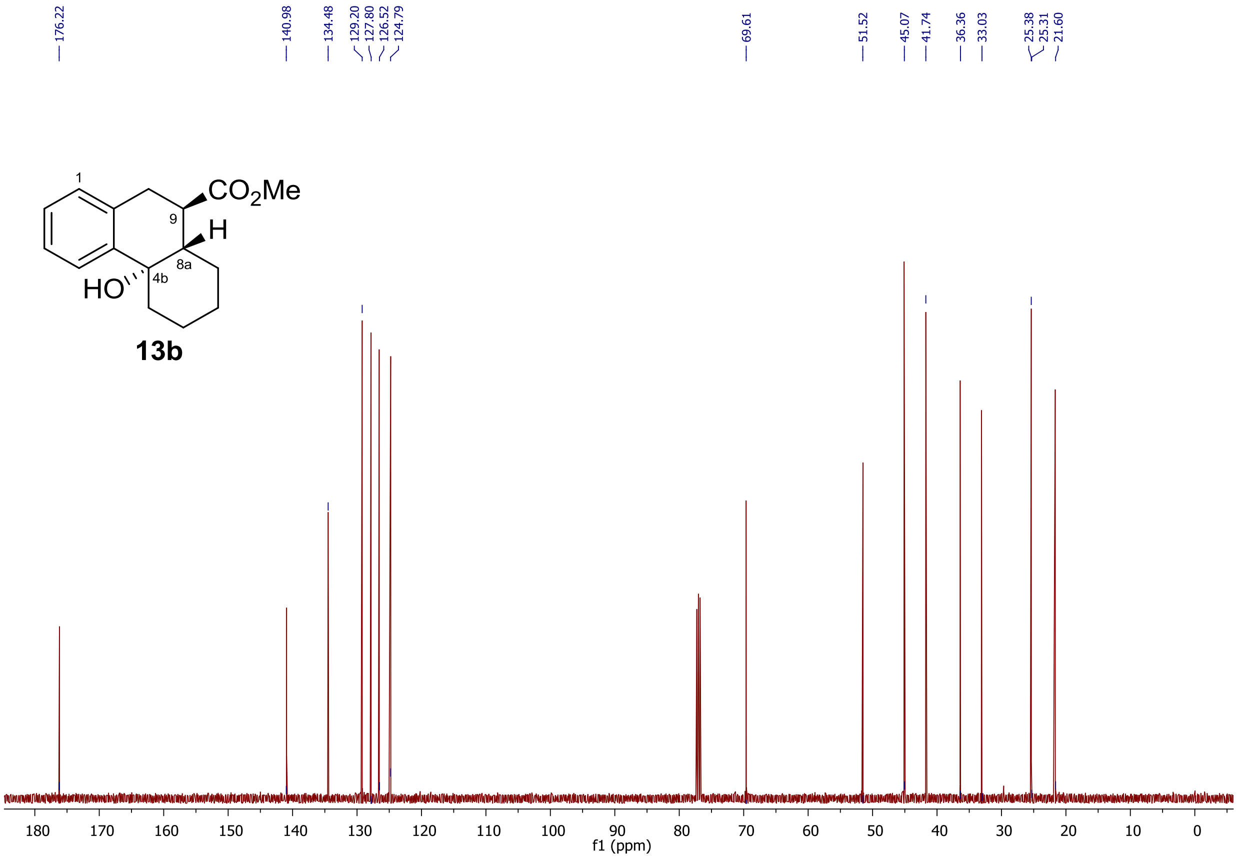
176.22
140.98
134.48
129.20
127.80
126.52
124.79
69.61
51.52
45.07
41.74
36.36
33.03
25.38
25.31
21.60
CO2Me
H
HO
1
9
8a
4b
13b
180
170
160
150
140
130
120
110
100
90
80
70
60
50
40
30
20
10
0
f1 (ppm)

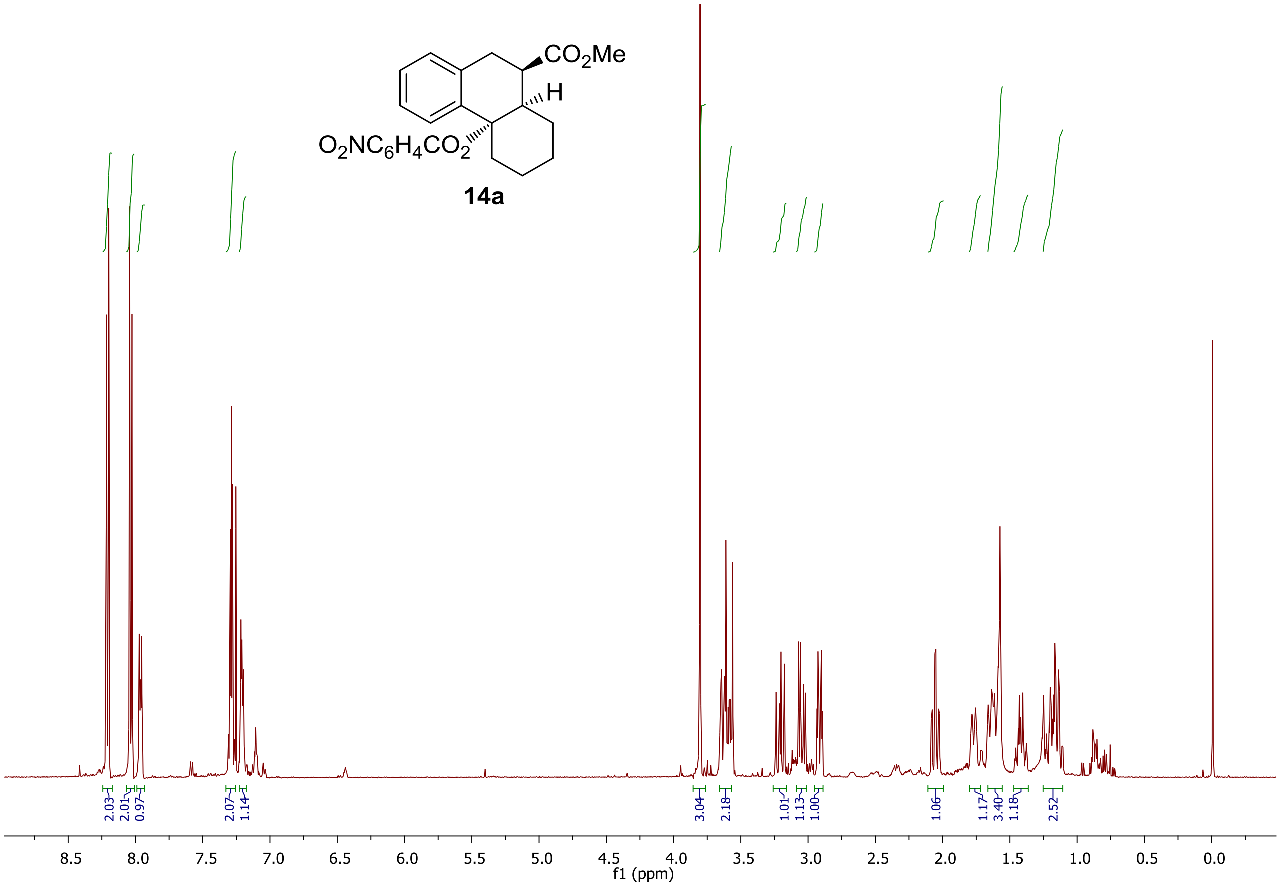
CO2Me
H
O2NC6H4CO2
14a
2.03
2.01
0.97
2.07
1.14
3.04
2.18
1.01
1.13
1.00
1.06
1.17
3.40
1.18
2.52
8.5
8.0
7.5
7.0
6.5
6.0
5.5
5.0
4.5
4.0
3.5
3.0
2.5
2.0
1.5
1.0
0.5
0.0
f1 (ppm)

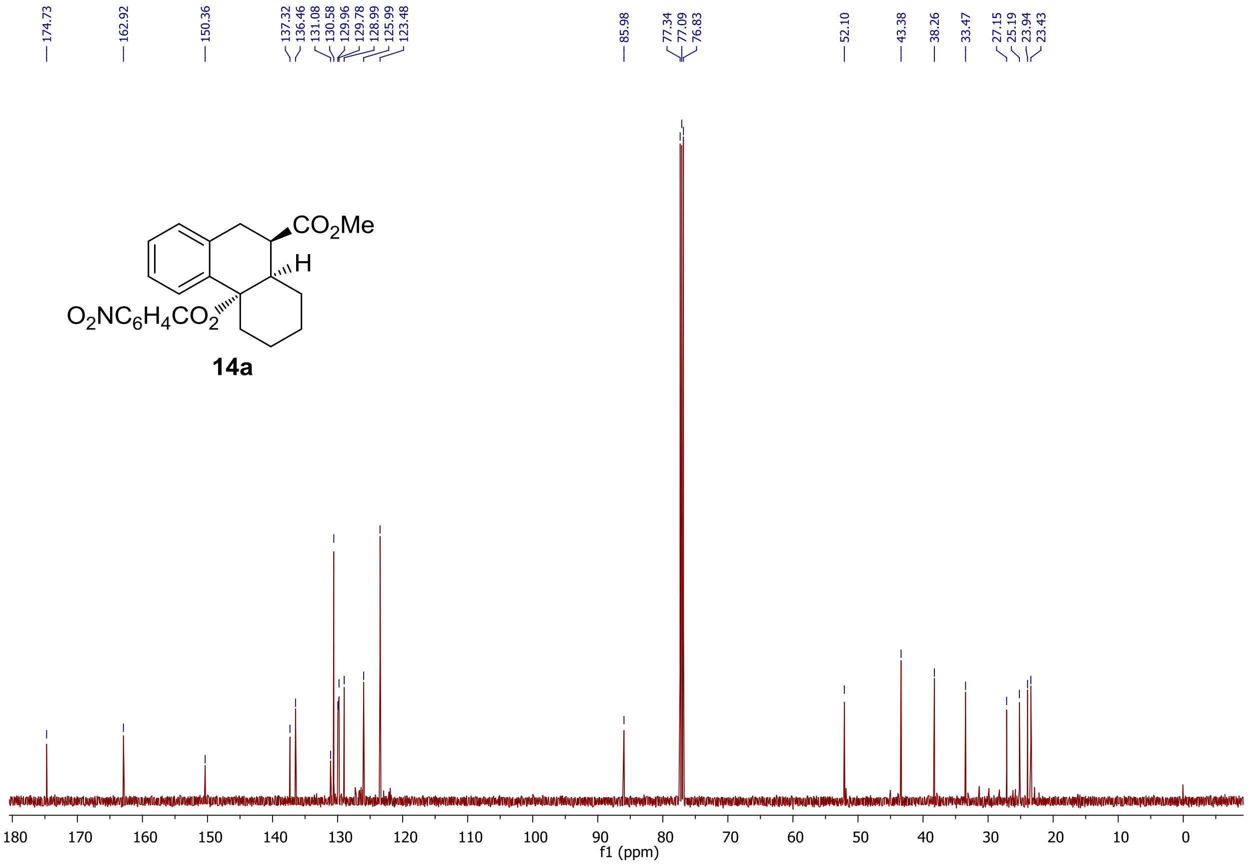
174.73
162.92
150.36
137.32
136.46
131.08
130.58
129.96
129.78
128.99
125.99
123.48
85.98
77.34
77.09
76.83
52.10
43.38
38.26
33.47
27.15
25.19
23.94
23.43
CO_2Me
H
$O_2NC_6H_4CO_2$
14a
180 170 160 150 140 130 120 110 100 90 80 70 60 50 40 30 20 10 0
f1 (ppm)

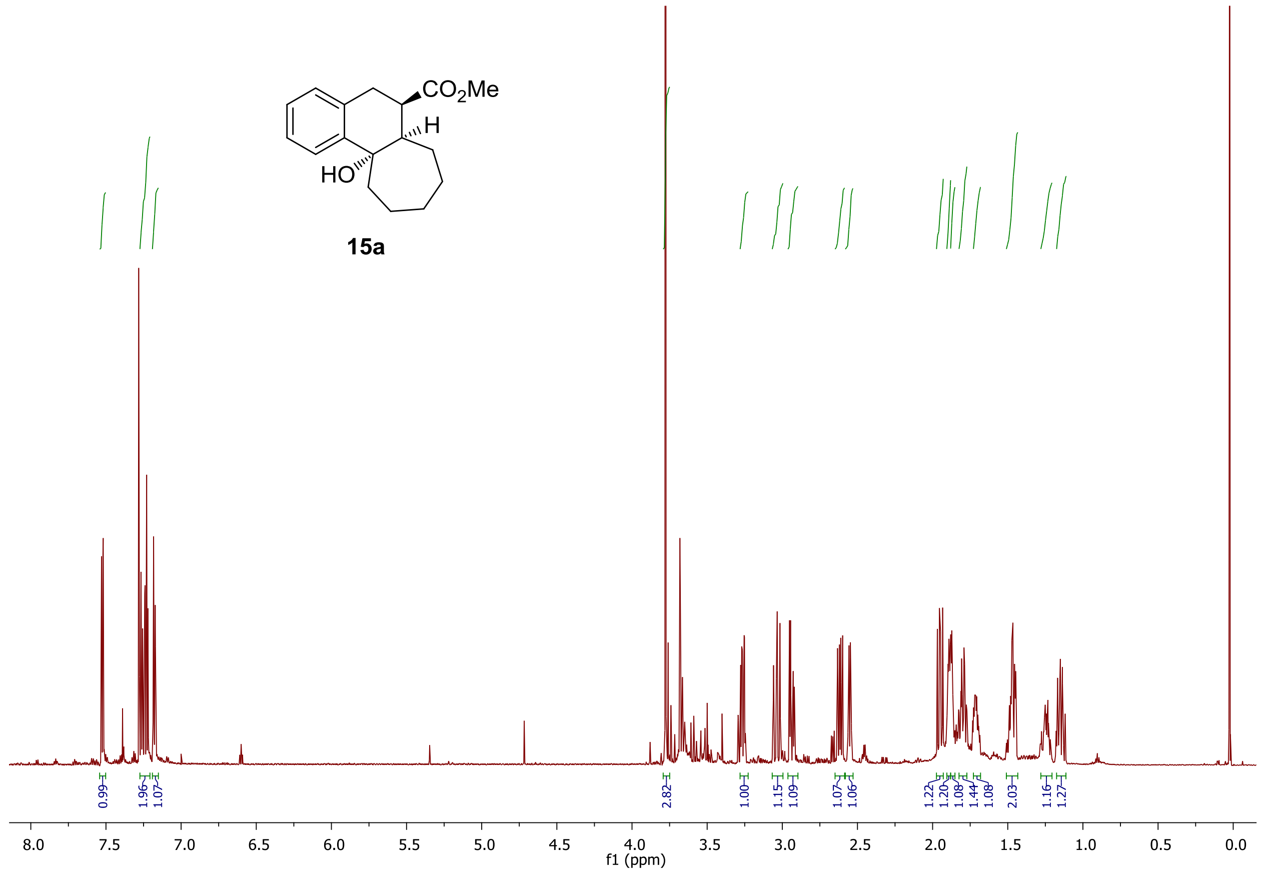
CO_2Me
H
HO
15a
0.99
1.96
1.07
2.82
1.00
1.15
1.09
1.07
1.06
1.22
1.20
1.08
1.44
1.08
2.03
1.16
1.27
8.0 7.5 7.0 6.5 6.0 5.5 5.0 4.5 4.0 3.5 3.0 2.5 2.0 1.5 1.0 0.5 0.0
f1 (ppm)

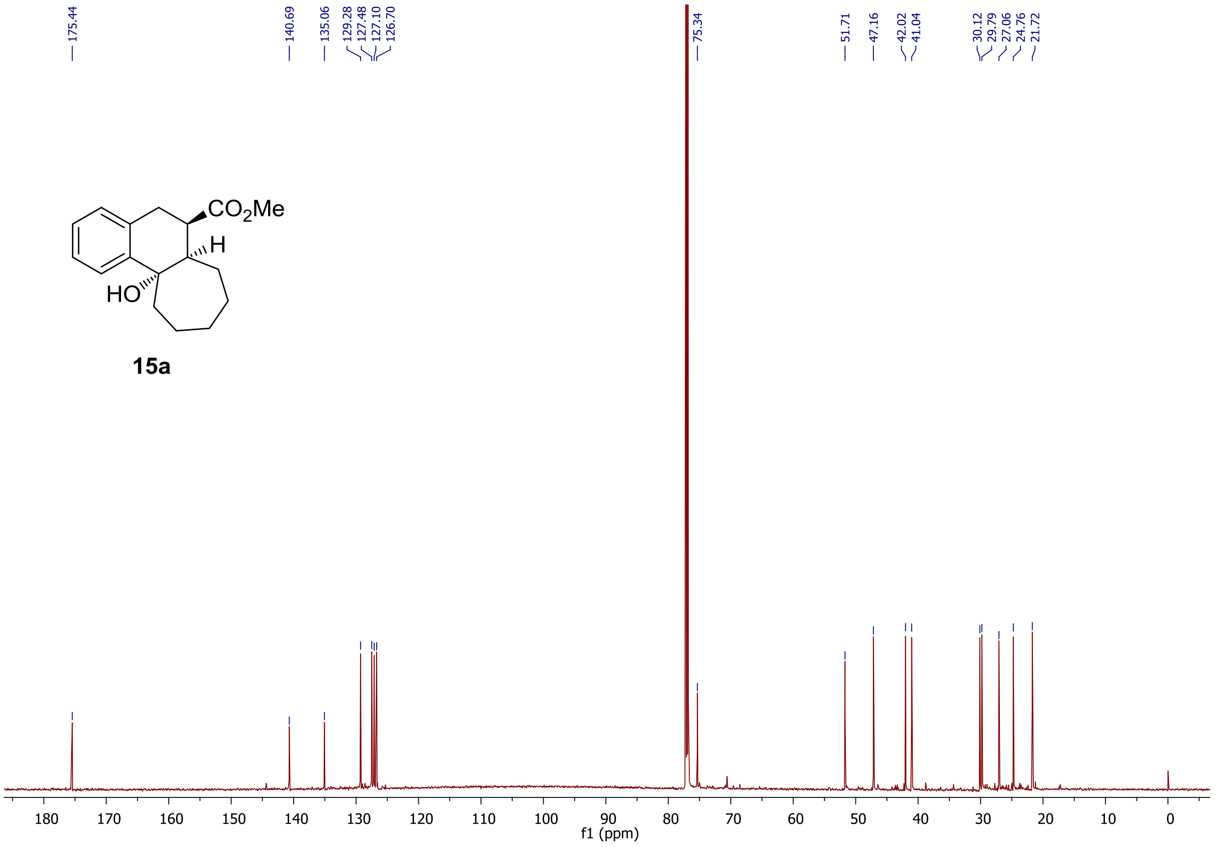
175.44
140.69
135.06
129.28
127.48
127.10
126.70
75.34
51.71
47.16
42.02
41.04
30.12
29.79
27.06
24.76
21.72
CO2Me
H
HO
15a
180 170 160 150 140 130 120 110 100 90 80 70 60 50 40 30 20 10 0
f1 (ppm)

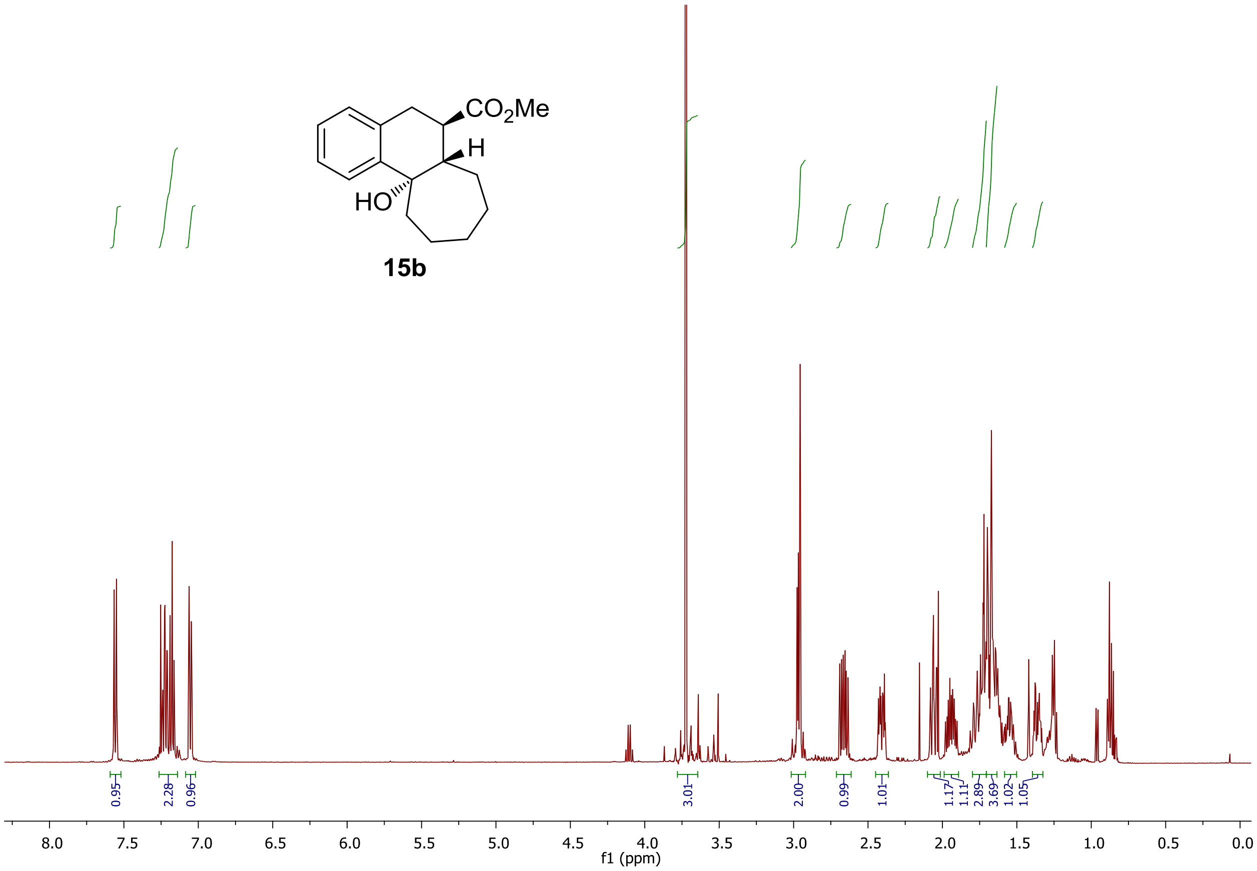
CO2Me
H
HO
15b
0.95
2.28
0.96
3.01
2.00
0.99
1.01
1.17
1.11
2.89
3.69
1.02
1.05
8.0 7.5 7.0 6.5 6.0 5.5 5.0 4.5 4.0 3.5 3.0 2.5 2.0 1.5 1.0 0.5 0.0
f1 (ppm)

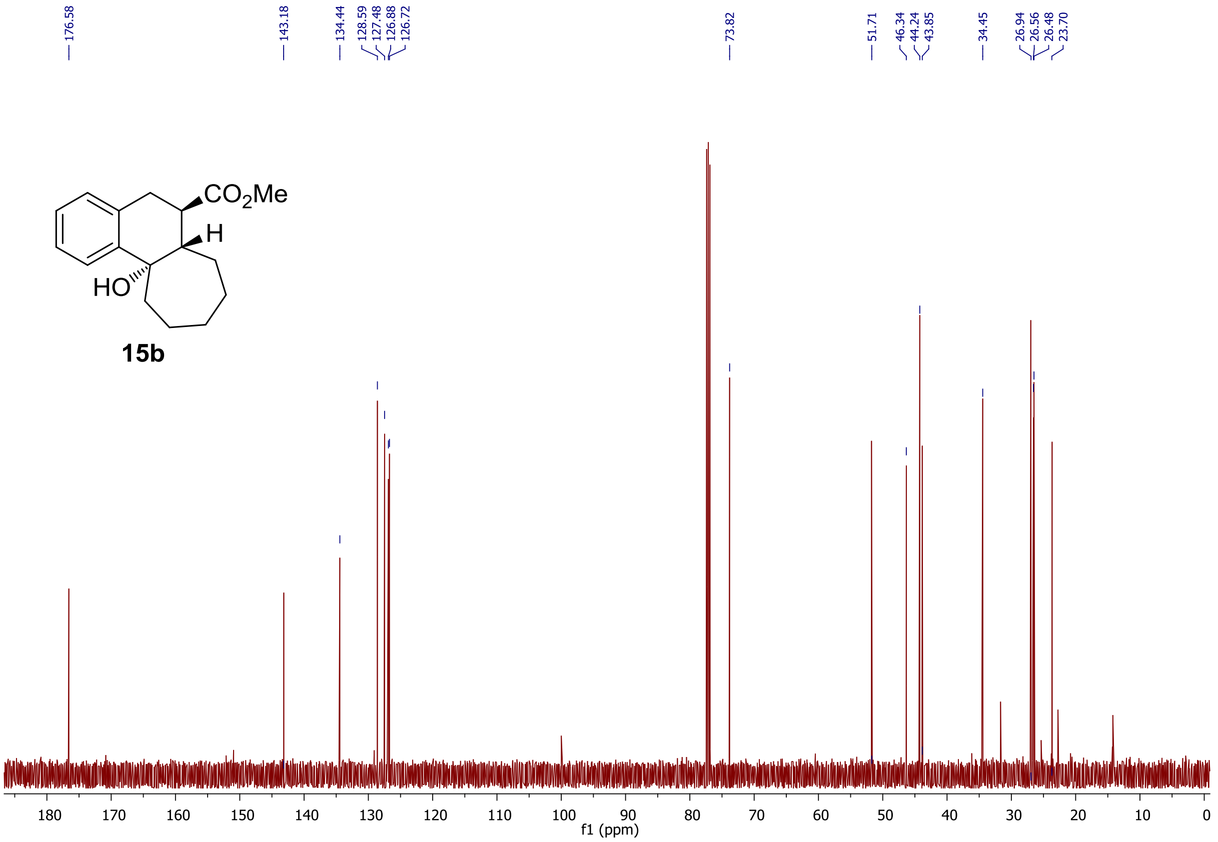
176.58
143.18
134.44
128.59
127.48
126.88
126.72
73.82
51.71
46.34
44.24
43.85
34.45
26.94
26.56
26.48
23.70
CO2Me
H
HO
15b
f1 (ppm)

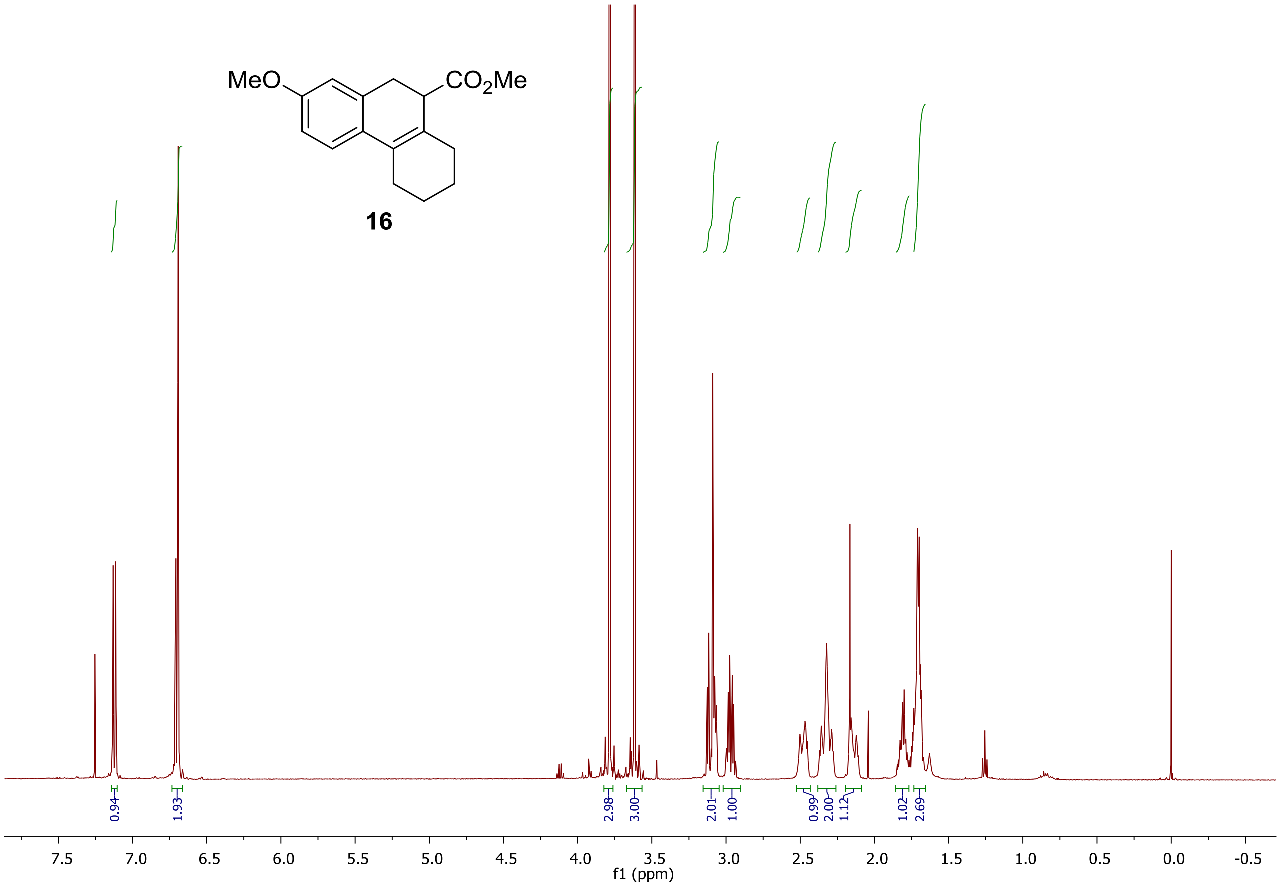
MeO
CO2Me
16
0.94
1.93
2.98
3.00
2.01
1.00
0.99
2.00
1.12
1.02
2.69
f1 (ppm)

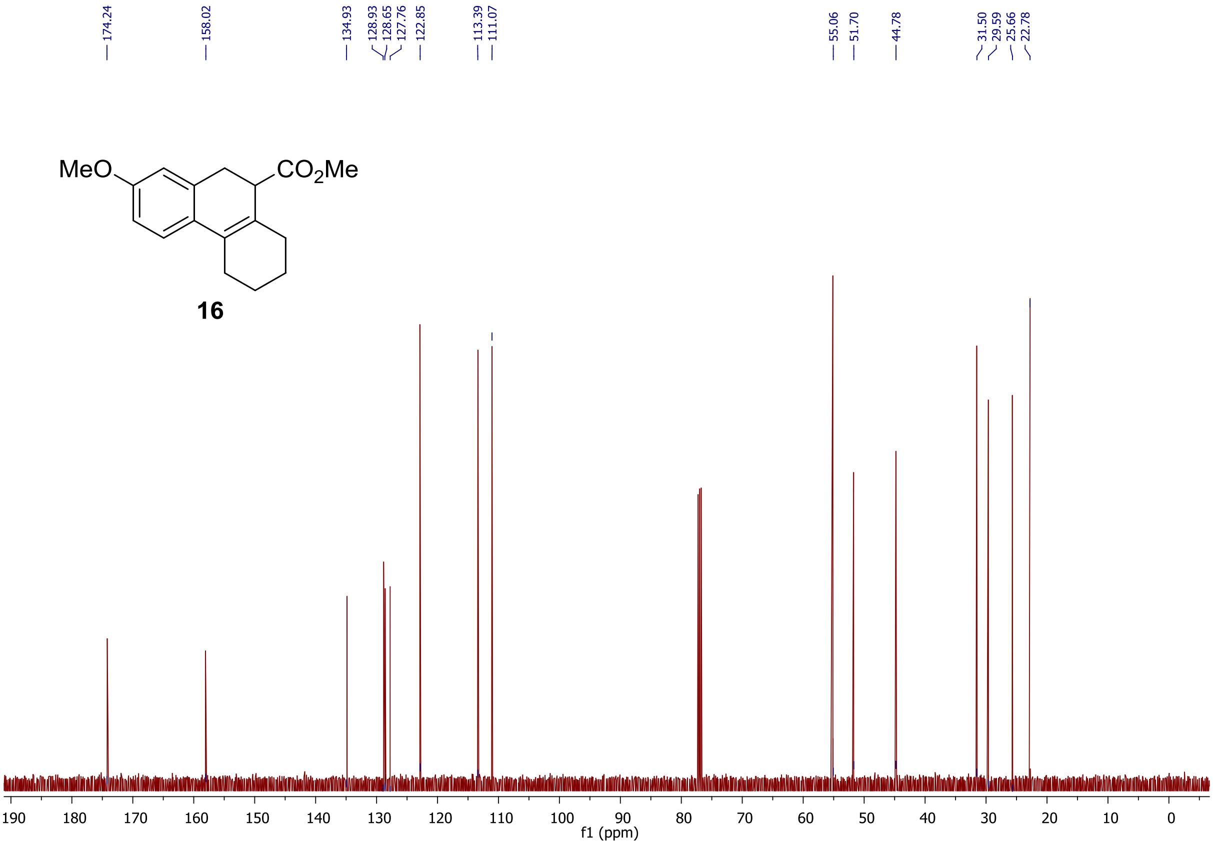
174.24
158.02
134.93
128.93
128.65
127.76
122.85
113.39
111.07
55.06
51.70
44.78
31.50
29.59
25.66
22.78
MeO
CO2Me
16
f1 (ppm)
